# Fast and precise single-cell data analysis using a hierarchical autoencoder

**DOI:** 10.1038/s41467-021-21312-2

**Published:** 2021-02-15

**Authors:** Duc Tran, Hung Nguyen, Bang Tran, Carlo La Vecchia, Hung N. Luu, Tin Nguyen

**Affiliations:** 1 Department of Computer Science and Engineering, University of Nevada Reno, Reno, NV USA; 2 Department of Clinical Sciences and Community Health, University of Milan, Milan, Italy; 3 Division of Cancer Control and Population Sciences, Hillman Cancer Center, University of Pittsburgh Medical Center, Pittsburgh, PA USA; 4 Department of Epidemiology, University of Pittsburgh Graduate School of Public Health, Pittsburgh, PA USA

**Keywords:** Machine learning, Software, RNA sequencing

## Abstract

A primary challenge in single-cell RNA sequencing (scRNA-seq) studies comes from the massive amount of data and the excess noise level. To address this challenge, we introduce an analysis framework, named single-cell Decomposition using Hierarchical Autoencoder (scDHA), that reliably extracts representative information of each cell. The scDHA pipeline consists of two core modules. The first module is a non-negative kernel autoencoder able to remove genes or components that have insignificant contributions to the part-based representation of the data. The second module is a stacked Bayesian autoencoder that projects the data onto a low-dimensional space (compressed). To diminish the tendency to overfit of neural networks, we repeatedly perturb the compressed space to learn a more generalized representation of the data. In an extensive analysis, we demonstrate that scDHA outperforms state-of-the-art techniques in many research sub-fields of scRNA-seq analysis, including cell segregation through unsupervised learning, visualization of transcriptome landscape, cell classification, and pseudo-time inference.

## Abstract (web summary)

Accurate analysis of single-cell RNA sequencing (scRNA-seq) data is affected by issues including technical noise and high dropout rate. Here, the authors develop a hierarchical autoencoder, scDHA, which outperforms existing methods in scRNA-seq analyses such as cell segregation and classification.

## Introduction

Advances in microfluidics and sequencing technologies have allowed us to monitor biological systems at single-cell resolution^1,2^. This comprehensive decomposition of complex tissues holds enormous potential in both developmental biology and clinical research^3–5^. Many computational methods have been developed to extract valuable information available in massive single-cell RNA sequencing data. These include methods for cell segregation, transcriptome landscape visualization, cell classification, and pseudo-time inference.

Defining cell types through unsupervised learning, also known as cell segregation or clustering, is considered the most powerful application of scRNA-seq data^6^. This has led to the creation of a number of atlas projects^7,8^, which aim to build the references of all cell types in model organisms at various developmental stages. Widely-used methods in this category include SC3^9^, SEURAT^10^, SINCERA^11^, CIDR^12^, and SCANPY^13^. Another fundamental application of scRNA-seq is the visualization of transcriptome landscape. Computational methods in this category aim at representing the high-dimensional scRNA-seq data in a low-dimensional space while preserving the relevant structure of the data. Non-linear methods^14^, including Isomap^15^, Diffusion Map^16^, t-SNE^17^, and UMAP^18^, have been recognized as efficient techniques to avoid overcrowding due to the large number of cells, while preserving the local data structure. Among these, t-SNE is the most commonly used technique while UMAP and SCANPY are recent methods.

Visualizing transcriptome landscape and building comprehensive atlases are problems of unsupervised learning. Once the cellular subpopulations have been determined and validated, classification techniques can be used to determine the composition of new data sets by classifying cells into discrete types. Dominant classification methods include XGBoost^19^, Random Forest (RF)^20^, Deep Learning (DL)^21^, and Gradient Boosting Machine (GBM)^22^. Another important downstream analysis is pseudo-time inference. Cellular processes, such as cell cycle, proliferation, differentiation, and activation^23,24^, can be modeled computationally using trajectory inference methods. These methods aim at ordering the cells along developmental trajectories. Among a number of trajectory inference tools, Monocle^25^, TSCAN^26^, Slingshot^27^, and SCANPY^13^ are considered state-of-the-art and are widely used for pseudo-temporal ordering.

As the volume of scRNA-seq data increases exponentially each year^28^, the above-mentioned methods have become primary investigation tools in many research fields, including cancer^29^, immunology^30^, or virology^31^. However, the ever-increasing number of cells, technical noise, and high dropout rate pose significant computational challenges in scRNA-seq analysis^6,32,33^. These challenges affect both analysis accuracy and scalability, and greatly hinder our capability to extract the wealth of information available in single-cell data.

**Fig. 1:**
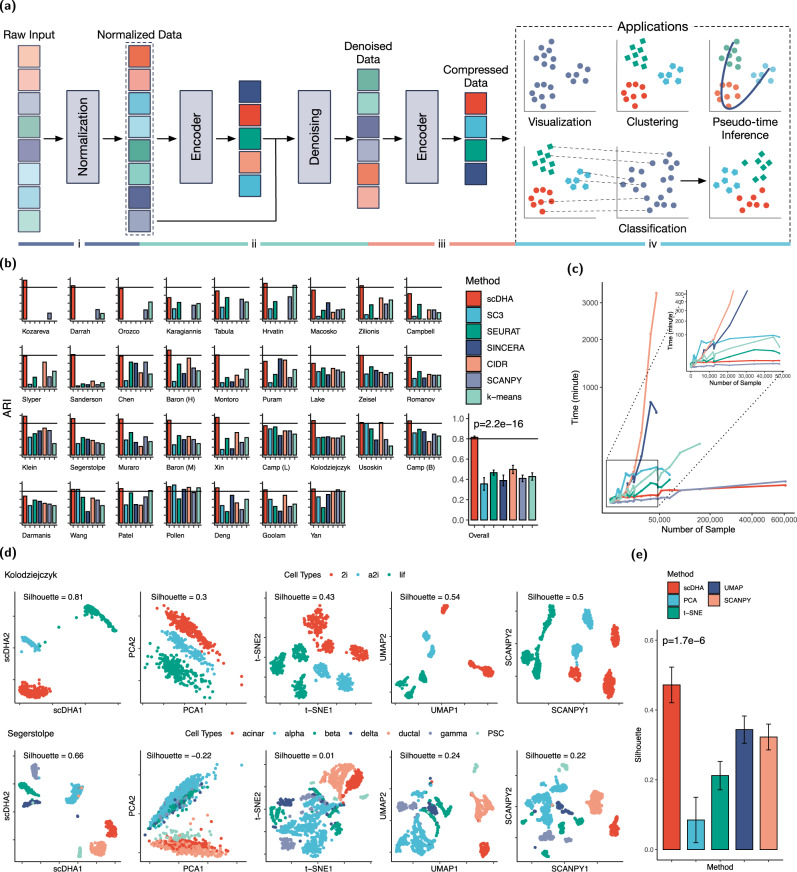
Overview of scDHA architecture and analysis performance on 34 scRNA-seq data sets. **a** Schematic overview of scDHA and applications: cell segregation through unsupervised learning, visualization, pseudo-temporal ordering, and cell classification. **b** Clustering performance of scDHA, SC3, SEURAT, SINCERA, CIDR, SCANPY, and k-means measured by adjusted Rand index (ARI). The first 34 panels show the ARI values obtained for individual data sets whereas the last panel shows the average ARIs and their variance (vertical segments). scDHA significantly outperforms other clustering methods by having the highest ARI values (*p* = 2.2 × 10^−16^ using one-sided Wilcoxon test). **c** Running time of the clustering methods, each using 10 cores. scDHA is the fastest among the six methods. **d** Color-coded representation of the Kolodziejczyk and Segerstolpe data sets using scDHA, PCA, t-SNE, UMAP, and SCANPY (from left to right). For each representation, we report the silhouette index, which measures the cohesion among cells of the same type, as well as the separation between different cell types. **e** Average silhouette values (bar plot) and their variance (vertical lines). scDHA significantly outperforms other dimension reduction methods by having the highest silhouette values (*p* = 1.7 × 10^−6^ using one-sided Wilcoxon test).

In this work, we develop a new analysis framework, called single-cell Decomposition using Hierarchical Autoencoder (scDHA), that can efficiently detach noise from informative biological signals. The scDHA pipeline consists of two core modules (Fig. 1a). The first module is a non-negative kernel autoencoder that provides a non-negative, part-based representation of the data. Based on the weight distribution of the encoder, scDHA removes genes or components that have insignificant contributions to the representation. The second module is a Stacked Bayesian Self-learning Network that is built upon the Variational Autoencoder (VAE)^34^ to project the data onto a low-dimensional space (see Methods section). Using this informative and compact representation, many analyses can be performed with high accuracy and tractable time complexity (mostly linear or lower complexity). In one joint framework, the scDHA software package conducts cell segregation through unsupervised learning, dimension reduction and visualization, cell classification, and time-trajectory inference. We will show that scDHA outperforms state-of-the-art methods in all four sub-fields: cell segregation through unsupervised learning, transcriptome landscape visualization, cell classification, and pseudo-time inference.

## Results

### Cell segregation

We assess the performance of scDHA in clustering using 34 scRNA-seq data sets with known cell types (see Methods section for details of each data set). The true class information of these data sets is only used a posteriori to assess the results. We compare scDHA with five methods that are widely used for single-cell clustering: SC3^9^, SEURAT^10^, SINCERA^11^, CIDR^12^, and SCANPY^13^. Note that SCANPY is also an all-in-one pipeline that is able to perform three types of analysis: clustering, visualization, and pseudo-time inference. We include k-means as the reference method in cluster analysis.

As the true cell types are known in these data sets, we use adjusted Rand index (ARI)^35^ to assess the performance of the six clustering methods. Figure 1b shows the ARI values obtained for each data set, as well as the average ARIs and their variances. scDHA outperforms all other methods by not only having the highest average ARI, but also being the most consistent method. The average ARI of scDHA across all 34 data sets is 0.81 with very low variability. The second best method, CIDR, has an average ARI of only 0.5. The one-sided Wilcoxon test also indicates that the ARI values of scDHA are significantly higher than the rest with a p-value of 2.2 × 10^−16^.

To perform a more comprehensive analysis, we calculate the normalized mutual information (NMI) and Jaccard index (JI) for each method (Supplementary Section 1 and Tables 2–4). We also compare the methods across different data platforms: plate-based, flow-cell-based, Smart-Seq1/2, SMARTer, inDrop, and 10X Genomics (see Supplementary Fig. 23). Regardless of the assessment metrics, scDHA consistently outperforms all other methods. At the same time, scDHA and SCANPY are the fastest among the seven methods (Fig. 1c and Supplementary Table 5). For the Macosko data set with 44 thousand cells, scDHA finishes the analysis in less than five minutes. On the contrary, it takes CIDR >2 days (3312 minutes) to finish the analysis of this data set. In summary, scDHA outperforms other clustering methods in terms of both accuracy and scalability.

We also assess the performance of the clustering methods using simulation. We use Splatter^36^ to generate 25 data sets with 10,000 genes and varying number of cells (5000, 10,000, 25,000, 50,000, and 100,000) and sparsity levels (28%, 32%, 37%, 44%, 51%). Supplementary Fig. 1 shows the ARI values obtained from comparing the discovered groups against the ground truth. Overall, scDHA has the highest ARI values in our analysis. Similar to the analysis of real data sets, scDHA and SCANPY are the fastest among the seven methods (see Supplementary Section 1.4 for more details).

**Fig. 2:**
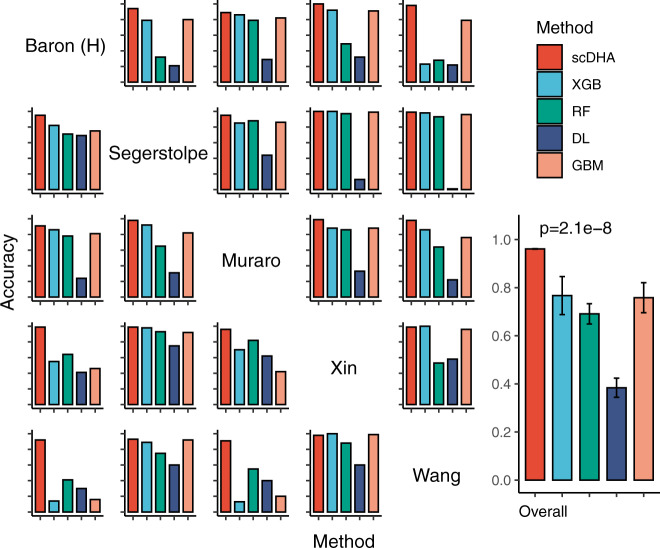
Classification accuracy of scDHA, XGBoost (XGB), Random Forest (RF), Deep Learning (DL), Gradient Boosted Machine (GBM) using five human pancreatic data sets. In each scenario (row), we use one data set as training and the rest as testing, resulting in 20 train-predict pairs. The overall panel shows the average accuracy values and their variance (vertical segment). The accuracy values of scDHA are significantly higher than those of other methods (*p* = 2.1 × 10^−8^ using Wilcoxon one-tailed test).

Note that the 34 single-cell data sets were normalized using different techniques by the data providers: raw counts (12 data sets), counts per million mapped reads (CPM, six data sets), reads per kilobase million (RPKM, eight data sets), and transcript per million (TPM, eight data sets). To understand the effect of normalization on the performance of scDHA, we re-normalize each data set using TPM, CPM, and RPKM, and then re-analyze the data. Our analysis shows that TMP-normalized data has a slight advantage over CPM- and RPKM-normalized data when using scDHA (see Supplementary Section 1.5 and Fig. 2).

### Dimension reduction and visualization

Here, we demonstrate that scDHA is more efficient than t-SNE, UMAP, and SCANPY, as well as the classical principal component analysis (PCA) in visualizing single-cell data. We test the five techniques on the same 34 single-cell data sets described above. Again, cell type information is not given as input to any algorithm.

The top row of Fig. 1d shows the color-coded representations of the Kolodziejczyk data set, which consists of three types of mouse embryo stem cells: *2i*, *a2i*, and *lif*. The classical PCA simply rotates the orthogonal coordinates to place dissimilar data points far apart in the two-dimensional (2D) space. In contrast, t-SNE focuses on representing similar cells together in order to preserve the local structure. In this analysis, t-SNE splits each of the two classes *2i* and *a2i* into two smaller groups, and *lif* class into three groups. The transcriptome landscape represented by UMAP is similar to that of t-SNE, in which UMAP also splits cells of the same type into smaller groups. According to the authors of this data set^37^, embryonic stem cells were cultured in three different conditions: *lif* (serum media that has leukemia inhibitory factor), *2i* (basal media that has GSK3*β* and Mek1/2 inhibitor), and *a2i* (alternative *2i* that has GSK3*β* and Src inhibitor). The *lif* cells were measured in two batches and both t-SNE and UMAP split this cell type according to batches. Similarly, the *a2i* cells were measured by two batches and the cells were separated according to batches. The *2i* cells were measured by four batches (chip1–2 cells, chip2–59 cells, chip3–72 cells, and chip4 - 82 cells). Both t-SNE and UMAP split the cells into two groups: the first group consists of cells from chip1 and the second group consists of cells from chip2, chip3, and chip4 (see Supplementary Section 2.2 and Fig. 18 for more details). SCANPY is able to mitigate batch effects in the *lif* cells but still splits *2i* and *a2i* cells. In contrast, scDHA provides a clear representation of the data, in which cells of the same type are grouped together and cells of different types are well separated.

The lower row of Fig. 1d shows the visualization of the Sergerstolpe data set (human pancreas). The landscapes of SCANPY, UMAP, and t-SNE are better than that of PCA. In these representations, the cell types are separable. However, the cells are overcrowded and many cells from different classes overlap. Also, the *alpha*, *beta,* and *gamma* cells are split into smaller groups. According to the authors of this data set^38^, the data were collected from different donors, which is potentially the source of heterogeneity. For this data set, scDHA better represents the data by clearly showing the transcriptome landscape with separable cell types.

To quantify the performance of each method, we calculate the silhouette index (SI)^39^ of each representation using true cell labels. This metric measures the cohesion among the cells of the same type and the separation among different cell types. For both data sets shown in Fig. 1d, the SI values of scDHA are much higher than those obtained for PCA, t-SNE, UMAP, and SCANPY. The visualization, SI values, and running time of all data sets are shown in Supplementary Fig. 9–17 and Tables 6 and 7. The average SI values obtained across the 34 data sets are shown in Fig. 1e. We also compare the methods across different data platforms: plate-based, flow-cell-based, Smart-Seq1/2, SMARTer, inDrop, and 10X Genomics (Supplementary Fig. 24). Overall, scDHA consistently and significantly outperforms other methods (*p* = 1.7 × 10^−6^).

### Cell classification

We assess scDHA’s classification capability by comparing it with four methods that are dominant in machine learning: XGBoost (XGB)^19^, Random Forest (RF)^20^, Deep Learning (DL)^21^, and Gradient Boosted Machine (GBM)^22^.

We test these methods using five data sets: Baron (8569 cells), Segerstolpe (2209 cells), Muraro (2126 cells), Xin (1600 cells), and Wang (457 cells). All five data sets are related to human pancreas and thus have similar cell types. In each analysis scenario, we use one data set as training and then classify the cells in the remaining four data sets. For example, we first train the models on Baron and then test them on Segerstolpe, Muraro, Xin, and Wang. Next, we train the models on Segerstolpe and test on the rest, etc. The accuracy of each method is shown in Fig. 2 and Supplementary Table 8.

Overall, scDHA is accurate across all 20 combinations with accuracy ranging from 0.88 to 1. scDHA outperforms other methods by having the highest accuracy. The average accuracy of scDHA is 0.96, compared with 0.77, 0.69, 0.43, and 0.72 for XGB, RF, DL, and GBM, respectively. In addition, scDHA is very consistent, while the performance of existing methods fluctuates from one analysis to another, especially when the testing data set is much larger than the training data set. For example, when the testing set (Baron) is 20 times larger than the training set (Wang), the accuracy of existing methods is close to 30%, whereas scDHA achieves an accuracy of 0.93. The one-sided Wilcoxon test also confirms that the accuracy values of scDHA are significantly higher than the rest (*p* = 2.1 × 10^−8^). Regarding time complexity, scDHA is the fastest with an average running time of two minutes per analysis (Supplementary Fig. 20).

### Time-trajectory inference

Here we compare the performance of scDHA with state-of-the-art methods for time-trajectory inference: Monocle^25^, TSCAN^26^, Slingshot^27^, and SCANPY^13^. We test scDHA and these methods using three mouse embryo development data sets: Yan, Goolam, and Deng. The true developmental stages of these data sets are only used a posteriori to assess the performance of the methods.

**Fig. 3:**
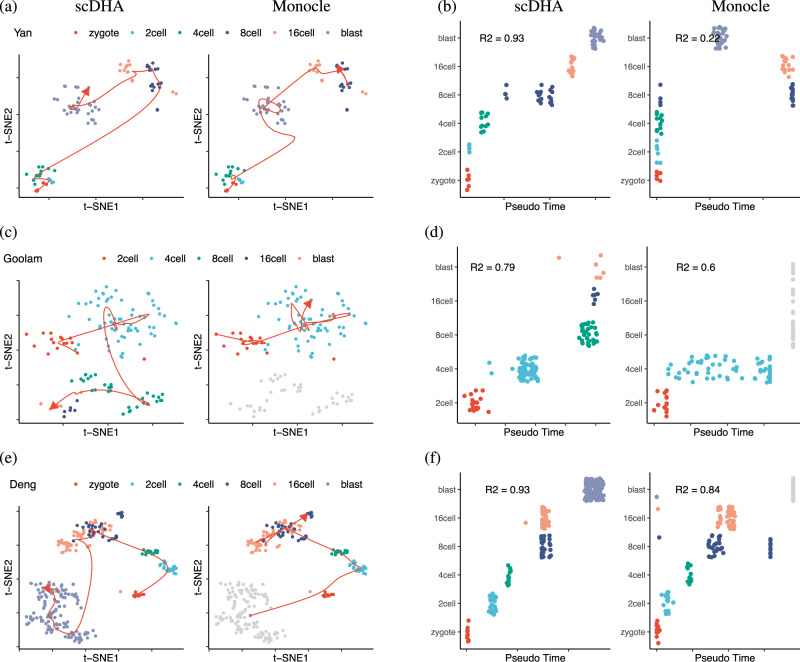
Pseudo-time inference of three mouse embryo development data sets (Yan, Goolam, and Deng) using scDHA and Monocle. **a** Visualized time-trajectory of the Yan data set in the first two t-SNE dimensions using scDHA (left) and Monocle (right). **b** Pseudo-temporal ordering of the cells in the Yan data set. The horizontal axis shows the inferred time for each cell while the vertical axis shows the true developmental stages. **c**, **d** Time-trajectory of the Goolam data set. Monocle is unable to estimate the time for most cells in 8cell, 16cell, and blast (colored in gray). **e**, **f** Time-trajectory of the Deng data set. Monocle is unable to estimate the pseudo-time for most blast cells.

Figure 3a shows the Yan data set in the first two t-SNE components. The smoothed lines shown in each panel indicate the time-trajectory of scDHA (left) and Monocle (right). The trajectory inferred by scDHA accurately follows the true developmental stages: it starts from zygote, going through 2cell, 4cell, 8cell, 16cell, and then stops at the blast class. On the contrary, the trajectory of Monocle goes directly from zygote to 8cell before coming back to 2cell. Figure 3b shows the cells ordered by pseudo-time. The time inferred by scDHA is strongly correlated with the true developmental stages. On the other hand, Monocle fails to differentiate between zygote, 2cell, and 4cell. To quantify how well the inferred trajectory explains the developmental stages, we also calculate the R-squared value. scDHA outperforms Monocle by having a higher R-squared value (0.93 compared with 0.84).

Figure 3c, d show the results of the Goolam data set. scDHA correctly reconstructs the time-trajectory whereas Monocle fails to estimate pseudo-time for 8cell, 16cell, and blast cells (colored in gray). Monocle assigns an "infinity” value for these cell classes. Figure 3e, f show the results obtained for the Deng data set. Similarly, the time-trajectory inferred by scDHA accurately follows the developmental stages, whereas Monocle cannot estimate the time for half of the cells. The results of TSCAN, Slingshot, and SCANPY are shown in Supplementary Fig. 21, 22. scDHA outperforms all three methods by having the highest R-squared values in every single analysis.

## Discussion

The ever-increasing number of cells, technical noise, and high dropout rate pose significant computational challenges in scRNA-seq analysis. These challenges affect both analysis accuracy and scalability, and greatly hinder our capability to extract the wealth of information available in single-cell data. To detach noise from informative biological signals, we have introduced scDHA, a powerful framework for scRNA-seq data analysis. We have shown that the framework can be utilized for both upstream and downstream analyses, including de novo clustering of cells, visualizing the transcriptome landscape, classifying cells, and inferring pseudo-time. We demonstrate that scDHA outperforms state-of-the-art techniques in each research sub-field. Although we focus on single-cell as an example, scDHA is flexible enough to be adopted in a range of research areas, from cancer to obesity to aging to any other area that employs high-throughput data.

In contrast to existing autoencoders, such as scVI^40^ that was developed for data imputation, scDHA provides a complete analysis pipeline from feature selection (first module) to dimension reduction (second module) and downstream analyses (visualization, clustering, classification, and pseudo-time inference). The scVI package itself is not capable of clustering, visualization, classification, and pseudo-time inference. Even for the implementation of autoencoder, there are two key differences between scDHA and scVI. First, scDHA implements a hierarchical autoencoder that consists of two modules: the first autoencoder to remove noise (denoising), and the second autoencoder to compress data. The added denoising module (first module) filters out the noisy features and thus improves the quality of the data. Second, we modify the standard VAE (second module) to generate multiple realizations of the input. This step makes the VAE more robust. Indeed, our analysis results show that scDHA and its second module consistently outperform scVI when scVI is used in conjunction with downstream analysis methods implemented in scDHA and other packages (see Supplementary Section 6 and Fig. 25–32).

In summary, scDHA is user-friendly and is expected to be more accurate than existing autoencoders. Users can apply scDHA to perform downstream analyses without installing additional packages for the four analysis applications (clustering, visualization, classification, and pseudo-time-trajectory inference). At the same time, the hierarchical autoencoder and the modified VAE (second module of scDHA) are expected to be more efficient than other autoencoders in single-cell data analysis.

## Methods

### Data and pre-processing

The 34 single-cell data sets used in our data analysis are described in Table 1. The data sets Montoro, Sanderson, Slyper, Zilionis, Karagiannis, Orozco, and Kozareva were downloaded from Broad Institute Single Cell Portal. The data sets Puram, Hrvatin, and Darrah were downloaded from Gene Expression Omnibus. Tabula Muris was downloaded from Figshare. The remaining 23 data sets were downloaded from Hemberg Group’s website (see Supplementary Table 1 for link to each data set). We removed samples with ambiguous labels from these data sets. Specifically, we removed cells with label “zothers” from Chen, “Unknown” from Camp (Brain), “dropped” from Wang, and “not applicable” from Segerstolpe. The only processing step we did was to perform log transformation (base 2) to rescale the data if the range of the data is larger than 100.

**Table 1:** Description of the 34 single-cell data sets used to assess the performance of computational methods.

Data set	Tissue	Size	Class	Protocol	Accession ID	Reference
1. Yan	Human embryo	90	6	Tang	GSE36552	Yan et al., 2013^50^
2. Goolam	Mouse embryo	124	5	Smart-Seq2	E-MTAB-3321	Goolam et al., 2016^51^
3. Deng	Mouse embryo	268	6	Smart-Seq2	GSE45719	Deng et al., 2014^52^
4. Pollen	Human tissues	301	11	SMARTer	SRP041736	Pollen et al., 2014^53^
5. Patel	Human tissues	430	5	Smart-Seq	GSE57872	Patel et al., 2014^4^
6. Wang	Human pancreas	457	7	SMARTer	GSE83139	Wang et al., 2016^54^
7. Darmanis	Human brain	466	9	SMARTer	GSE67835	Darmanis et al., 2015^55^
8. Camp (Brain)	Human brain	553	5	SMARTer	GSE75140	Camp et al., 2015^56^
9. Usoskin	Mouse brain	622	4	STRT-Seq	GSE59739	Usoskin et al., 2015^57^
10. Kolodziejczyk	Mouse embryo stem cells	704	3	SMARTer	E-MTAB-2600	Kolodziejczyk et al., 2015^37^
11. Camp (Liver)	Human liver	777	7	SMARTer	GSE81252	Camp et al., 2017^58^
12. Xin	Human pancreas	1,600	8	SMARTer	GSE81608	Xin et al., 2016^59^
13. Baron (Mouse)	Mouse pancreas	1,886	13	inDrop	GSE84133	Baron et al., 2016^60^
14. Muraro	Human pancreas	2,126	10	CEL-Seq2	GSE85241	Muraro et al., 2016^61^
15. Segerstolpe	Human pancreas	2,209	14	Smart-Seq2	E-MTAB-5061	Segerstolpe et al., 2016^38^
16. Klein	Mouse embryo stem cells	2,717	4	inDrop	GSE65525	Klein et al., 2015^62^
17. Romanov	Mouse brain	2,881	7	SMARTer	GSE74672	Romanov et al., 2017^63^
18. Zeisel	Mouse brain	3,005	9	STRT-Seq	GSE60361	Zeisel et al., 2015^3^
19. Lake	Human brain	3,042	16	Fluidigm C1	phs000833.v3.p1	Lake et al., 2016^64^
20. Puram	Human tissues	5,902	10	Smart-Seq2	GSE103322	Puram et al., 2017^65^
21. Montoro	Human pancreas	7,193	7	Smart-Seq2	GSE103354	Montoro et al., 2018^66^
22. Baron (Human)	Human pancreas	8,569	14	inDrop	GSE84133	Baron et al., 2016^60^
23. Chen	Mouse brain	12,089	46	Drop-seq	GSE87544	Chen et al., 2017^67^
24. Sanderson	Mouse tissues	12,648	11	10X Genomics	SCP916	Sanderson et al., 2020^68^
25. Slyper	Human blood	13,316	8	10X Genomics	SCP345	
26. Campbell	Mouse brain	21,086	21	Drop-seq	GSE93374	Campbell et al., 2017^69^
27. Zilionis	Human lung	34,558	9	inDrop	GSE127465	Zilionis et al., 2019^70^
28. Macosko	Mouse retina	44,808	12	Drop-seq	GSE63473	Macosko et al., 2015^71^
29. Hrvatin	Mouse visual cortex	48,266	8	inDrop	GSE102827	Hrvatin et al., 2018^72^
30. Tabula Muris	Mouse tissues	54,439	40	10X Genomics	GSE109774	Schaum et al., 2018^73^
31. Karagiannis	Human blood	72,914	12	10X Genomics	GSE128879	Karagiannis et al., 2020^74^
32. Orozco	Human eye	100,055	11	10X Genomics	GSE135133	Orozco et al., 2020^75^
33. Darrah	Human blood	162,490	14	Drop-seq	GSE139598	Darrah et al., 2020^76^
34. Kozareva	Mouse cerebellum	611,034	18	10X Genomics	SCP795	Kozareva et al., 2020^77^

The first two columns describe the name and tissue while the next five columns show the number of cells, number of cell types, protocol, accession ID, and reference.

### Software package and setting

In our analysis, we followed the instruction and tutorials provided by the authors of each software package. We used the default parameters of each tool to perform the analysis. The memory limit for all analysis methods is set to 200GB of RAM.

For clustering, we compared scDHA with SC3, SEURAT, SINCERA, CIDR, SCANPY and k-means. We used the following packages: (i) SC3 version 1.10.1 from Bioconductor, (ii) SEURAT version 2.3.4 from CRAN, (iii) CIDR version 0.1.5 from GitHub (github.com/VCCRI/CIDR), (iv) scanpy version 1.4.4 from Anaconda, (v) SINCERA script provided by Hemberg group (scrnaseq-course.cog.sanger.ac.uk/website/biological-analysis.html), and (vi) stats for k-means in conjunction with PCA implementation available in the package irlba version 2.3.3 from CRAN. For k-means, we used the first 100 principal components for clustering purpose. In contrast to the other five methods, k-means cannot determine the number of clusters. Therefore, we also provided the true number of cell types for k-means. In addition, since k-means often converges to local optima, we ran k-means using 1000 different sets of starting points and then chose the partitioning with the smallest squared error.

For dimension reduction and visualization, we used the following packages: (i) irlba version 2.3.3 from CRAN for PCA, (ii) Rtsne version 0.15 from CRAN for t-SNE, (iii) scanpy version 1.4.4, and (iv) python package umap-learn version 0.3.9 from Anaconda python distribution for UMAP. This python package is run through a wrapper in R package umap version 0.2.2.

For classification, we compared scDHA with XGBoost, RF, DL, and GBM. We used the R package H2O version 3.24.0.5 from CRAN. This package provides the implementation of XGBoost, RF, DL, and GBM. All models were run with fivefold cross-validation for better accuracy.

For time-trajectory inference, we compared scDHA with Monocle, TSCAN, Slingshot, and SCANPY. We used the following packages: (i) R package Monocle3 version 0.1.1 from GitHub (github.com/cole-trapnell-lab/monocle3), (ii) TSCAN version 1.20.0 from Bioconductor, (iii) Slingshot version 1.3.1 from Bioconductor, and (iv) scanpy version 1.4.4.

### scDHA pipeline

scDHA requires an expression matrix *M* as input, in which rows represent cells and columns represent genes or transcripts. Given the input *M*, scDHA automatically performs a log transformation (base 2) to rescale the data if the range of *M* is higher than 100. The goal is to prevent the domination of genes or features with high expression.

scDHA pipeline for scRNA sequencing data analysis consists of two core modules (Figure 1a). The first module is a non-negative kernel autoencoder that provides a non-negative, part-based representation of the data. Based on the weight distribution of the encoder, scDHA removes genes or components that have insignificant contributions to the part-based representation. The second module is a Stacked Bayesian Self-learning Network that is built upon the VAE^34^ to project the data onto a low-dimensional space. For example, for clustering application, the first module automatically rescales the data and removes genes with insignificant contribution to the part-based representation. The second module then projects the clean data to a low-dimensional latent space using VAE before separating the cells using k-nearest neighbor spectral clustering. The details of each step are described below.

### Non-negative kernel autoencoder

To reduce the technical variability and heterogeneous calibration from sequencing technologies, the expression data are rescaled to a range of 0 to 1 for each cell as follow:

1 $${X}_{ij}=\frac{{M}_{ij}-min({M}_{i.})}{max({M}_{i.})-min({M}_{i.})}$$

where *M* is the input matrix and *X* is the normalized matrix. This min-max scaling step is to reduce standard deviation and to suppress the effect of outliers, which is frequently used in DL models^41,42^ (see Supplementary Section 1.6 and Fig. 3 for more details).

After normalization, the data are then passed through a one-layer autoencoder to filter out insignificant genes/features. In short, autoencoder consists of two components: encoder and decoder. The formulation of autoencoder can be written as follows:

2 $$e	={f}_{E}(x)\\ \bar{x}	={f}_{D}(e)$$

where $x\in {R}_{+}^{n}$ is the input of the model (x is simply a row/sample, i.e., *x* = *X*_*i*._), *f*_*E*_ and *f*_*D*_ represent the transformation by encoder and decoder layers, $\bar{x}$ is the reconstruction of *x*. The encoder and decoder transformations can be represented as *f*_*E*_(*x*) = *xW*_*E*_ + *b*_*E*_ and *f*_*D*_(*e*) = *eW*_*D*_ + *b*_*D*_, where *W*-s are the weight matrices and *b*-s are the bias vectors. Encoder aims at representing the data in a much lower dimensional space (compression) whereas decoder tries to reconstruct the original input from the compressed data. Optimizing this process can theoretically result in a compact representation of the original, high-dimensional data. The size of the bottleneck layer is set to 50 nodes (not user-provided parameter). Changing this number of nodes has no significant impact on the results of scDHA (see Supplementary Fig. 5).

**Fig. 4:**
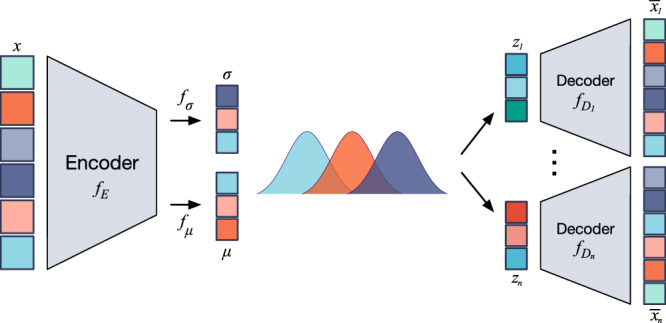
High-level representation of stacked Bayesian autoencoder. The encoder projects input data to multiple low-dimensional latent spaces (outputs of *z*_1_ to *z*_*n*_ layers). The decoders infer original data from these latent data. Minimizing the difference between inferred data and original one leads to a high quality representation of the original data at bottleneck layer (outputs of *μ* layer).

In our model, the weights of the encoder (*W*_*E*_ in *f*_*E*_(⋅)) are forced to be non-negative so that each latent variable is an additive combination of the original features. By doing so, the non-negative coefficients of the less important features will be shrunk toward zero (see Supplementary Section 1.7 and Fig. 4 for more discussion). Based on the computed weights, the method only keeps genes or components with high weight variances. In principle, the set of these genes can be considered a “sufficient and necessary” set to represent the original data. These genes are necessary because removing them would greatly damage the reversibility of the decoder, i.e., decoder cannot accurately reconstruct the original data. At the same time, they are sufficient because the encoder automatically shrinks the weights of genes or gene groups that have similar but lesser impacts in the compression procedure. By default, scDHA selects 5000 genes but users can choose a different number based on the weight distribution (see Supplementary Section 1.8 and Fig. 6)

### Stacked Bayesian autoencoder

After the gene filtering step using non-negative kernel autoencoder, we obtain a data matrix in which each gene is considered critical to preserve cell heterogeneity. However, although the step has greatly reduced the number of features, the number of genes is still in the scale of hundreds or thousands. Therefore, it is necessary to perform dimension reduction before conducting any analysis or visualization. For this purpose, we developed a modified version of VAE (theorized by Kingma et al.^34^). We name it stacked Bayesian autoencoder (Figure 4) since the model is designed with multiple latent spaces, instead of only one latent space used in the original VAE or any other autoencoder model.

VAE has the same basic structure as a standard autoencoder, which is a self-learning model consisting of two components: encoder and decoder. Given the input matrix (the filtered matrix obtained from Non-negative kernel autoencoder), VAE’s encoder constructs a low-dimensional representation of the input matrix while the decoder aims at inferring the original data. By minimizing the difference between the inferred and the input data, the middle bottleneck layer is considered as the “near lossless” projection of the input onto a latent space with a low number of dimensions (*m* = 15 by default). We keep the model size small to avoid overfitting and force the neuron network to be as compressed as possible. Also, restricting the size of the latent layer will converge cells from the same group into similar latent space manifold. At the same time, the size of the latent layer needs to be sufficient (15 dimensions) to keep the latent variables disentangled. Per our experience, varying *m* between 10 and 20 does not alter the analysis results.

Given an expression profile of a cell *x*, the formulation of this architecture can be formulated as follows:

3 $$e	={f}_{E}(x)\\ \mu 	={f}_{\mu }(e)\\ \sigma 	={f}_{\sigma }(e)\\ z 	\sim N(\mu ,{\sigma }^{2})\\ \bar{x}	={f}_{D}(z)$$

where $x\in {R}_{+}^{n}$ is the input of the network, *f*_*E*_ and *f*_*D*_ represent the transformation by encoder and decoder layers. In addition to the standard autoencoder, two transformations *f*_*μ*_ and *f*_*σ*_ are added on the output *e* of encoder to generate the parameters *μ* and *σ* (*μ*, *σ* ∈ *R*^*m*^). The compressed data *z* is now sampled from the distribution *N*(*μ*, *σ*^2^). In contrast to the standard autoencoder, VAE uses *z* as the input of the decoder instead of *e*. By adding randomness in generating *z*, VAE prevents overfitting by avoiding mapping the original data to the compressed space without learning a generalized representation of data. The perturbation process was shown to be an effective method to increase data stability^43^.

In our stacked model, to further diminish overfitting and increase the robustness, we generate multiple compressed spaces with multiple realizations of *z*. For that purpose, we use a re-parameterization trick to generate multiple realizations of *z* as follows: *z* = *μ* + σ∗*N*(0, 1). This re-parameterization trick is introduced to ensure that the model can backpropagate^34^.

To train our model, we use AdamW^44^ as optimizer while adopting a two-stage training scheme^45^: (i) a warm-up process, which uses only reconstruction loss, and (ii) the VAE stage, in which the Kullback–Leibler loss is also considered to ensure the normal distribution of latent variables *z*. The warm-up process prevents the model from ignoring reconstruction loss and only focuses on Kullback–Leibler loss. By doing this, we avoid the pitfall of making the model fail to learn generalized representations of the data. This process also makes the model less sensitive to the weight initialization. For faster convergence and better accuracy, scaled exponential linear unit^46^ is used as the activation function.

After finishing the training stage, the input data are processed through the encoder to generate representative latent variables of original data. This compressed representation of the data will be used for single-cell applications: (1) cell segregation through unsupervised learning, (2) transcriptome landscape visualization, (3) pseudo-time-trajectory inference, and (4) cell classification.

### Cell segregation via clustering

#### Predicting the number of cell types

The number of cell types is determined using two indices: (i) the ratio of between sum of squares over the total sum of squares, and (ii) the increase of the within sum of squares when the number of clusters increases. The indices are formulated as follows:

4 $$Index\,1=\frac{S{S}_{between,j}}{S{S}_{total,j}}$$

5 $$Index\,2=\frac{S{S}_{within,j+1}-S{S}_{within,j}}{S{S}_{within,j}}$$

where *j* is the number of clusters.

Larger *Index 1* means that members of one group are far from other groups, i.e., the clusters are well separated. *Index 2* is affected by the number of eigenvectors generated by spectral decomposition, which is also the number of clusters. We assume that the addition of an eigenvector that leads to the highest spike in the within sum of squares (which is undesirable) would be the correct number of clusters. These indices are calculated by performing k-nearest neighbor spectral clustering on a subset of samples over a range of cluster numbers. Mean of the predictions from these two indices is set to be the final number of clusters (see Supplementary Fig.

#### Basic clustering algorithm

In order to improve the accuracy when clustering non-spherical data while ensuring the fast running time, we apply a k-nearest neighbor adaption of spectral clustering (k-nn SC) as the clustering method embedded in our package. Instead of using Euclidean distance to determine the similarity between two samples, Pearson correlation is used to improve the stability of cluster assignment. The difference between k-nn SC and normal SC is that the constructed affinity matrix of data points is sparse. For each data point, the distance is calculated for only its k-nearest neighbors while the distance to the rest is left at zero. The clustering process of k-nn SC consists of four steps: (i) constructing affinity matrix *A* for all data points to use as input graph, (ii) generating a symmetric and normalized Laplacian matrix ${L}^{\text{sym}}=I-{D}^{-\frac{1}{2}}A{D}^{-\frac{1}{2}}$ where *D* is the degree matrix of the graph, *A* is the constructed affinity matrix and *I* is the identity matrix, (iii) calculating eigenvalues for Laplacian matrix and select those with smallest values, generating eigenvectors corresponding to selected eigenvalues, (iv) performing final clustering using k-means on the obtained eigenvectors.

#### Consensus clustering

We use the basic clustering algorithm described above to cluster the compressed data. To achieve higher accuracy and to avoid local minima, an ensemble of data projection models is used. We first repeat the data projection and clustering process multiple times. We then combine the clustering results using the Weighted-based meta-clustering (wMetaC) implemented in SHARP^47^. wMetaC is conducted through five steps: (i) calculating cell–cell weighted similarity matrix *W*, *w*_*i*,*j*_ = *s*_*i*,*j*_(1 − *s*_*i*,*j*_) where *s*_*i*,*j*_ is the chance that cell *i* and *j* are in the same cluster, (ii) calculating cell weight, which is the sum of all cell–cell weights related to this cell, (iii) generating cluster-cluster similarity matrix ∣*C*∣*x*∣*C*∣, where C is the union of all the clusters obtained in each replicate, (iv) performing hierarchical clustering on cluster-cluster similarity matrix, and (v) determining final results by a voting scheme.

#### Voting procedure

**Fig. 5:**
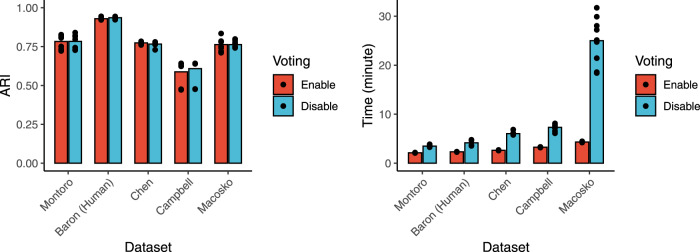
Accuracy and running time of scDHA on large data sets with and without using the voting procedure. The voting procedure significantly reduces the running time without compromising the accuracy. Each point represents the result of a single run, while the bar shows the average of 10 runs.

For large data sets, we also provide an additional option in our package to reduce the time complexity without compromising the performance. Instead of clustering the whole data set, which requires a large amount of memory and heavy computation, we can perform the clustering on a subset of the data points and then apply a vote-counting procedure to assign the rest of the data to each cluster. The voting process is based on the k-nearest neighbor classification. This approach still ensures the high clustering quality without compromising the speed of the method, as shown in Figure 5.

#### Dimension reduction and visualization

Given the compressed data (10–15 dimensions), we compute the distance matrix for the cells and then perform log and *z* transformations as follows:

6 $${D}_{ij}=\frac{\mathrm{log}\,({D}_{ij})-{\mu }_{\mathrm{log}\,({D}_{i.})}}{{\sigma }_{\mathrm{log}\,({D}_{i.})}}$$

where *D* is a distance matrix. The rationale of this transformation is to make the distribution of distances from one point to its neighbors more uniform. Next, we calculate the probabilities *p*_*ij*_ that are proportional to the similarity between sample *i* and *j* as follows:

7 $${p}_{j| i}=\frac{\exp ({D}_{ij})}{{\sum }_{k\ne i}\exp ({D}_{ik})}$$

At the same time, using the compressed data, we build a neural network to project the data to two-dimensional space. Using two formulas described above, we re-calculate the probabilities *q*_*ij*_ that are proportional to the similarity between sample *i* and *j* in the two-dimensional space. Our goal is to learn a two-dimensional projection of the data that retains the probabilities *p* as well as possible. We achieve this by minimizing the distance between *Q* and *P*. Here, we use the Kullback–Leibler divergence to represent the distance between the two probability distributions, which can be formulated as:

8 $$KL(P| | Q)={\sum }_{i\ne j}{p}_{ij}\mathrm{log}\,\frac{{p}_{ij}}{{q}_{ij}}$$

By minimizing Kullback–Leibler divergence, we obtain the optimal representation of the data in the two-dimensional space. The algorithm can be generalized to three or higher number of dimensions.

### Classification

The problem can be described as follows. We are given two data sets of the same tissue: the training data set and the testing data set. For the training data set, we have the cell labels. The goal is to determine the cell labels of the testing data set.

Our classification procedure consists of the following steps: (i) concatenate the two matrices into a single matrix, in which the rows consist of all cells from the two data sets and columns are the common genes; (ii) normalize and compress the merged data using the hierarchical autoencoder described above; (iii) compute the similarity matrix for the cells using Pearson correlation; and finally (iv) determine the label of cells from testing data using k-nearest neighbor algorithm (k-nn).

The rationale for concatenating the two data sets is to exploit the robust denoising and dimension reduction procedure offered by the hierarchical autoencoder. Since we normalize the data per each cell, different scaling of the two data sets (training or testing) would not pose as a problem. At the same time, the hierarchical autoencoder efficiently diminishes batch effect and noise, moving cells of the same type closer to one another. We demonstrated that even with an unsophisticated classification technique as k-nn, scDHA is proven to be better than current state-of-the-art methods, including XGBoost, RF, DL, and GBM.

### Time-trajectory inference

We implement a pseudo-time inference method that allows users to infer non-branching trajectory that is correlated with the developmental stages of cells. This method requires a starting point as part of the input. We note that users can easily apply any other methods on the compressed data provided by scDHA (see Saelens et al.^48^ for a comprehensive list of pseudo-time inference methods). Given the compressed data, our method computes the similarity distance for the cells using Pearson correlation. Using this similarity matrix as the affinity matrix, we construct a graph in which nodes represent cells and edges represent the distance between the cells. In order to construct the pseudo-time trajectory, we apply the minimum spanning tree (MST) algorithm on the graph to find the shortest path that goes through all cells. From the MST, pseudo-time is determined by distance from one point to the designated starting point.

### Statistics and reproducibility

The scDHA package is installed in the docker image that is available at http://scdha.tinnguyen-lab.com/, which has all tools, dependencies, and scripts so that readers can reproduce all results. All analyses are performed with fixed random seed to ensure reproducibility.

### Reporting summary

Further information on research design is available in the Nature Research Reporting Summary linked to this article.

## Supplementary information

Supplementary Information

Reporting Summary

## Data Availability

The details of 34 single-cell data sets analyzed in the article can be found in Table 1. The links to publicly available sources are reported in Supplementary Table 1. The processed data can also be found at http://scdha.tinnguyen-lab.com/.

## Code availability

The scDHA package^49^ is available as an independent software at https://github.com/duct317/scDHA.
