# Supplementary material for: Fast and precise single-cell data analysis using a hierarchical autoencoder
Source: Nat Commun. 2021 Feb 15;12:1029. doi: 10.1038/s41467-021-21312-2 (PMC7884436; doi:10.1038/s41467-021-21312-2)
Supplement: Supplementary file 1 — Supplementary Information [file 41467_2021_21312_MOESM1_ESM.pdf]

Fast and precise single-cell data analysis using hierarchical autoencoder

Supplementary Information

Duc Tran[1], Hung Nguyen[1], Bang Tran[1], Carlo La Vecchia[2], Hung N. Luu[3,4], and Tin Nguyen[1,*]

[1] Department of Computer Science and Engineering, University of Nevada Reno, Reno, NV, USA
[2] Department of Clinical Sciences and Community Health, University of Milan, Milan, Italy
[3] Division of Cancer Control and Population Sciences, Hillman Cancer Canter, University of Pittsburgh Medical Center, Pittsburgh, PA, USA
[4] Department of Epidemiology, University of Pittsburgh Graduate School of Public Health, Pittsburgh, PA, USA
* tinn@unr.edu

Contents

1 Supplementary Note 1: Unsupervised clustering of single-cell data **3**
1.1 Evaluation metrics . . . 3
1.2 Data availability . . . 3
1.3 Performance evaluation . . . 3
1.4 Simulation study . . . 9
1.5 Impact of scRNA-seq normalization . . . 10
1.6 Impact of min-max scaling on scDHA . . . 10
1.7 Impact of non-negative constraint on scDHA . . . 10
1.8 Impact of model parameters on scDHA . . . 11
1.9 Predicting the number of cell types for Klein dataset . . . 12

2 Supplementary Note 2: Dimension reduction and visualization **15**
2.1 Performance evaluation . . . 15
2.2 Batch effects in the Kolodziejczyk dataset . . . 27

3 Supplementary Note 3: Cell classification **29**

4 Supplementary Note 4: Time trajectory inference **31**

5 Supplementary Note 5: Effects of data platforms on single-cell analysis **33**

6 Supplementary Note 6: Impact of the hierarchical autoencoder **35**

References **41**

1 Supplementary Note 1: Unsupervised clustering of single-cell data

1.1 Evaluation metrics

We use three different metrics to assess the performance of the six clustering methods: adjusted Rand index (ARI), Normalized Mutual Information (NMI), and Jaccard index.

Rand index (RI)[1] measures the agreement between a given clustering and the ground truth. RI is calculated as:

$$RI = \frac{a+b}{a+b+c+d} = \frac{a+b}{\binom{N}{2}} \tag{1}$$

where a is the number of pairs that belong to the same true cell type and are clustered together, b is the number of pairs that belong to different true cell types and are not clustered together, c is the number of pairs that belong to the same cell types and are not clustered together, d is the number of pairs that belong to different cell types and are clustered together, and $\binom{N}{2}$ is the number of possible pairs that can be formed from the N cells. Intuitively, RI is the fraction of pairs that are grouped in the same way (either together or not) in the two partitions compared (e.g. 0.9 means 90% of pairs are grouped in the same way). The adjusted Rand index (ARI)[2] is the corrected-for-chance version of the Rand Index. The ARI takes values from -1 to 1, with the ARI expected to be 0 for a random subtyping.

Jaccard index (JI) is also known as Intersection over Union. In our context, The Jaccard index is basically the number of pairs that belong to the same true cell type and are clustered together, divided by the number of pairs that are either in the same true cell type or are clustered together. JI is calculated as:

$$J = \frac{a}{a+b+c} \tag{2}$$

Finally, Normalized Mutual Information (NMI) is a normalized version of Mutual Information (MI). Denoting X as the true labeling of the cells and Y is the partitioning obtained from a clustering method, the NMI is calculated as:

$$NMI = \frac{1}{2} \times \frac{I(X;Y)}{H(X)+H(Y)} \tag{3}$$

where $I(X;Y)$ is the mutual information between X and Y. $H(X)$ is the entropy of the true partition X and $H(Y)$ is the entropy of the partition obtained from clustering. The NMI value take a range from 0 to 1 in which 1 indicates a perfect match between cell types and clusters. In contrast, 0 value means no mutual information between cell types and clusters.

1.2 Data availability

We downloaded 34 scRNA-seq datasets from public repositories. The datasets Montoro, Sanderson, Slyper, Zilionis, Karagiannis, Orozco, Kozareva were downloaded from Broad Institute Single Cell Portal. The datasets Puram, Hrvatin, and Darrah were downloaded from Gene Expression Omnibus. Tabula Muris was downloaded from Figshare. The remaining 23 datasets were downloaded from Hemberg Group's website. Supplementary Table 1 shows the specific link to each of the 34 datasets.

1.3 Performance evaluation

We assess the performance of the six clustering methods, scDHA, SC3[3], SEURAT[4], SINCERA[5], CIDR[6], SCANPY, and k-means, using ARI, NMI and JI described above. In our analysis, we limit the memory

usage to 200 GB of RAM. SINCERA and CIDR crashed when analyzing datasets with 48,266 cells (Hrvatin) or larger. SC3 and SEURAT ran out of memory when analyzing datasets with 100,055 cells (Orozco) or larger. *k-means* ran out of memory when analyzing the Kozareva dataset with 611,034 cells. Supplementary Table 2 shows the ARI values while Supplementary Tables 3 and 4 shows the NMI and JI values, respectively. Regardless of the evaluation metrics, scDHA consistently outperforms other six methods.

Supplementary Table 1. Link to 34 single-cell datasets.

Dataset	Link
1. Yan	https://hemberg-lab.github.io/scRNA.seq.datasets/human/edev/
2. Goolam	https://hemberg-lab.github.io/scRNA.seq.datasets/mouse/edev/#goolam
3. Deng	https://hemberg-lab.github.io/scRNA.seq.datasets/mouse/edev/#deng
4. Pollen	https://hemberg-lab.github.io/scRNA.seq.datasets/human/tissues/#pollen
5. Patel	https://hemberg-lab.github.io/scRNA.seq.datasets/human/tissues/#patel
6. Wang	https://hemberg-lab.github.io/scRNA.seq.datasets/human/pancreas/#wang
7. Darmanis	https://hemberg-lab.github.io/scRNA.seq.datasets/human/brain/#darmanis
8. Camp (Brain)	https://hemberg-lab.github.io/scRNA.seq.datasets/human/brain/
9. Usoskin	https://hemberg-lab.github.io/scRNA.seq.datasets/mouse/brain/#usoskin
10. Kolodziejczyk	https://hemberg-lab.github.io/scRNA.seq.datasets/mouse/esc/#kolodziejczyk
11. Camp (Liver)	https://hemberg-lab.github.io/scRNA.seq.datasets/human/liver/
12. Xin	https://hemberg-lab.github.io/scRNA.seq.datasets/human/pancreas/#xin
13. Baron (Mouse)	https://hemberg-lab.github.io/scRNA.seq.datasets/mouse/pancreas/
14. Muraro	https://hemberg-lab.github.io/scRNA.seq.datasets/human/pancreas/#muraro
15. Segerstolpe	https://hemberg-lab.github.io/scRNA.seq.datasets/human/pancreas/#segerstolpe
16. Klein	https://hemberg-lab.github.io/scRNA.seq.datasets/mouse/esc/
17. Romanov	https://hemberg-lab.github.io/scRNA.seq.datasets/mouse/brain/#romanov
18. Zeisel	https://hemberg-lab.github.io/scRNA.seq.datasets/mouse/brain/#zeisel
19. Lake	https://hemberg-lab.github.io/scRNA.seq.datasets/human/brain/#lake
20. Puram	https://www.ncbi.nlm.nih.gov/geo/query/acc.cgi?acc=GSE103322
21. Montoro	https://www.ncbi.nlm.nih.gov/geo/query/acc.cgi?acc=GSE103354
22. Baron (Human)	https://hemberg-lab.github.io/scRNA.seq.datasets/human/pancreas/
23. Chen	https://hemberg-lab.github.io/scRNA.seq.datasets/mouse/brain/#chen
24. Sanderson	https://singlecell.broadinstitute.org/single_cell/study/SCP916/
25. Slyper	https://singlecell.broadinstitute.org/single_cell/study/SCP345/
26. Campbell	https://hemberg-lab.github.io/scRNA.seq.datasets/mouse/brain/
27. Zilionis	https://singlecell.broadinstitute.org/single_cell/study/SCP739/
28. Macosko	https://hemberg-lab.github.io/scRNA.seq.datasets/mouse/retina/
29. Hrvatin	https://www.ncbi.nlm.nih.gov/geo/query/acc.cgi?acc=GSE102827
30. Tabula Muris	https://doi.org/10.6084/m9.figshare.5968960.v3
31. Karagiannis	https://singlecell.broadinstitute.org/single_cell/study/SCP587/
32. Orozco	https://singlecell.broadinstitute.org/single_cell/study/SCP484/
33. Darrah	https://www.ncbi.nlm.nih.gov/geo/query/acc.cgi?acc=GSE139598
34. Kozareva	https://singlecell.broadinstitute.org/single_cell/study/SCP795/

Supplementary Table 2. Performance of scDHA, SC3, SEURAT, SINCERA, CIDR, SCANPY, and k-means on 34 single-cell datasets measured by adjusted Rand index (ARI). Cells with NA values indicate that the method was not able to analyze the dataset (crashed or out-of-memory). Cells highlighted in green have the highest ARI values. The average ARI of scDHA is 0.81, which is much higher than the rest (CIDR is the second best with an average ARI of 0.5). In addition, scDHA has the highest ARI values in all but two datasets (Pollen and Puram).

Dataset	Size	Class	scDHA	SC3	SEURAT	SINCERA	CIDR	SCANPY	k-means
1. Yan	90	6	0.86	0.66	0.39	0.72	0.80	0.84	0.80
2. Goolam	124	5	0.84	0.60	0.42	0.30	0.70	0.42	0.48
3. Deng	268	6	0.89	0.44	0.29	0.70	0.51	0.34	0.60
4. Pollen	301	11	0.92	0.96	0.61	0.85	0.90	0.77	0.89
5. Patel	430	5	0.87	0.46	0.76	0.47	0.45	0.66	0.82
6. Wang	457	7	0.85	0.85	0.65	0.29	0.63	0.58	0.44
7. Darmanis	466	9	0.68	0.44	0.58	0.55	0.50	0.48	0.44
8. Camp (B)	553	5	0.86	0.56	0.65	0.59	0.34	0.53	0.48
9. Usoskin	622	4	0.82	0.80	0.66	0.38	0.82	0.39	0.23
10. Kolodziejczyk	704	3	0.87	0.44	0.45	0.46	0.43	0.43	0.48
11. Camp (L)	777	7	0.74	0.66	0.71	0.49	0.61	0.61	0.53
12. Xin	1,600	8	0.95	0.14	0.42	0.16	0.57	0.32	0.44
13. Baron (M)	1,886	13	0.88	0.26	0.49	0.39	0.47	0.39	0.29
14. Muraro	2,126	10	0.91	0.38	0.57	0.32	0.22	0.46	0.34
15. Segerstolpe	2,209	14	0.92	0.29	0.44	0.40	0.37	0.31	0.29
16. Klein	2,717	4	0.98	0.45	0.54	0.61	0.68	0.62	0.29
17. Romanov	2,881	7	0.75	0.22	0.39	0.23	0.32	0.30	0.30
18. Zeisel	3,005	9	0.80	0.33	0.51	0.42	0.37	0.32	0.36
19. Lake	3,042	16	0.60	0.39	0.48	0.31	0.47	0.43	0.38
20. Puram	5,902	10	0.65	0.11	0.32	0.71	0.68	0.24	0.44
21. Montoro	7,193	7	0.81	0.11	0.24	0.13	0.30	0.20	0.45
22. Baron (H)	8,569	14	0.93	0.14	0.58	0.34	0.73	0.48	0.41
23. Chen	12,089	46	0.78	0.16	0.63	0.60	0.36	0.63	0.33
24. Sanderson	12,648	11	0.82	0.03	0.08	0.06	0.15	0.06	0.11
25. Slyper	13,316	8	0.78	0.07	0.25	0.00	0.63	0.26	0.40
26. Campbell	21,086	21	0.64	0.07	0.37	0.00	0.23	0.23	0.16
27. Zilionis	34,558	9	0.84	0.11	0.36	0.02	0.53	0.38	0.48
28. Macosko	44,808	12	0.73	0.07	0.22	0.41	0.17	0.23	0.25
29. Hrvatin	48,266	8	0.90	0.26	0.44	NA	NA	0.56	0.85
30. Tabula Muris	54,439	40	0.71	0.30	0.54	NA	NA	0.50	0.43
31. Karagiannis	72,914	12	0.54	0.26	0.42	NA	NA	0.35	0.39
32. Orozco	100,055	11	0.77	NA	NA	NA	NA	0.23	0.43
33. Darrah	162,490	14	0.84	NA	NA	NA	NA	0.24	0.14
34. Kozareva	611,034	18	0.98	NA	NA	NA	NA	0.15	NA
Mean ARI			0.81	0.36	0.47	0.39	0.50	0.41	0.43

Supplementary Table 3. Performance of scDHA, SC3, SEURAT, SINCERA, CIDR, SCANPY, and k-means on 34 single-cell datasets measured by normalized mutual information (NMI). Cells with NA values indicate that the method was not able to analyze the dataset (crashed or out-of-memory). Cells highlighted in green have the highest NMI values. scDHA outperforms other methods by having the highest average NMI value. In addition, scDHA has the highest NMI values in 31 out of 34 datasets.

Dataset	Size	Class	scDHA	SC3	SEURAT	SINCERA	CIDR	SCANPY	k-means
1. Yan	90	6	0.89	0.80	0.55	0.82	0.84	0.87	0.86
2. Goolam	124	5	0.82	0.80	0.61	0.61	0.78	0.71	0.63
3. Deng	268	6	0.89	0.73	0.53	0.73	0.74	0.70	0.78
4. Pollen	301	11	0.96	0.95	0.80	0.93	0.94	0.91	0.94
5. Patel	430	5	0.84	0.67	0.76	0.67	0.57	0.72	0.83
6. Wang	457	7	0.83	0.81	0.71	0.43	0.71	0.71	0.57
7. Darmanis	466	9	0.75	0.67	0.64	0.66	0.64	0.69	0.62
8. Camp (B)	553	5	0.82	0.68	0.70	0.62	0.49	0.69	0.55
9. Usoskin	622	4	0.81	0.79	0.74	0.54	0.80	0.65	0.31
10. Kolodziejczyk	704	3	0.90	0.68	0.68	0.54	0.57	0.67	0.51
11. Camp (L)	777	7	0.85	0.81	0.85	0.69	0.79	0.82	0.72
12. Xin	1,600	8	0.87	0.39	0.60	0.42	0.55	0.61	0.60
13. Baron (M)	1,886	13	0.85	0.65	0.75	0.61	0.51	0.74	0.59
14. Muraro	2,126	10	0.88	0.69	0.77	0.51	0.43	0.74	0.53
15. Segerstolpe	2,209	14	0.90	0.65	0.75	0.62	0.45	0.69	0.53
16. Klein	2,717	4	0.97	0.69	0.71	0.67	0.66	0.76	0.40
17. Romanov	2,881	7	0.69	0.43	0.60	0.31	0.34	0.58	0.35
18. Zeisel	3,005	9	0.78	0.62	0.67	0.47	0.47	0.63	0.55
19. Lake	3,042	16	0.67	0.68	0.73	0.47	0.54	0.73	0.62
20. Puram	5,902	10	0.79	0.45	0.66	0.68	0.63	0.62	0.63
21. Montoro	7,193	7	0.74	0.30	0.50	0.24	0.46	0.47	0.56
22. Baron (H)	8,569	14	0.88	0.50	0.80	0.46	0.72	0.77	0.63
23. Chen	12,089	46	0.77	0.53	0.79	0.53	0.42	0.77	0.63
24. Sanderson	12,648	11	0.71	0.21	0.43	0.29	0.12	0.40	0.40
25. Slyper	13,316	8	0.73	0.36	0.60	0.16	0.70	0.59	0.62
26. Campbell	21,086	21	0.68	0.49	0.74	0.15	0.38	0.69	0.48
27. Zilionis	34,558	9	0.83	0.41	0.70	0.08	0.58	0.66	0.62
28. Macosko	44,808	12	0.59	0.31	0.56	0.19	0.33	0.56	0.40
29. Hrvatin	48,266	8	0.92	0.59	0.74	NA	NA	0.77	0.88
30. Tabula Muris	54,439	40	0.80	0.65	0.77	NA	NA	0.77	0.68
31. Karagiannis	72,914	12	0.66	0.49	0.73	NA	NA	0.66	0.65
32. Orozco	100,055	11	0.76	NA	NA	NA	NA	0.60	0.70
33. Darrah	162,490	14	0.78	NA	NA	NA	NA	0.61	0.34
34. Kozareva	611,034	18	0.92	NA	NA	NA	NA	0.58	NA
Mean NMI			0.81	0.60	0.68	0.50	0.58	0.68	0.60

Supplementary Table 4. Performance of scDHA, SC3, SEURAT, SINCERA, CIDR, SCANPY, and k-means on 34 single-cell datasets measured by Jaccard Index (JI). Cells with NA values indicate that the method was not able to analyze the dataset (crashed or out-of-memory). Cells highlighted in green have the highest JI values. scDHA outperforms other methods by having the highest average JI value. scDHA also has the highest JI values in 31 out of 34 datasets.

Dataset	Size	Class	scDHA	SC3	SEURAT	SINCERA	CIDR	SCANPY	k-means
1. Yan	90	6	0.80	0.57	0.38	0.64	0.73	0.77	0.73
2. Goolam	124	5	0.82	0.54	0.46	0.28	0.65	0.37	0.45
3. Deng	268	6	0.86	0.40	0.33	0.65	0.46	0.28	0.55
4. Pollen	301	11	0.87	0.93	0.50	0.76	0.83	0.66	0.82
5. Patel	430	5	0.81	0.35	0.67	0.36	0.38	0.55	0.75
6. Wang	457	7	0.81	0.81	0.58	0.31	0.56	0.50	0.39
7. Darmanis	466	9	0.59	0.34	0.48	0.46	0.42	0.37	0.35
8. Camp (B)	553	5	0.82	0.48	0.57	0.55	0.30	0.44	0.44
9. Usoskin	622	4	0.76	0.74	0.58	0.35	0.78	0.31	0.29
10. Kolodziejczyk	704	3	0.83	0.37	0.38	0.44	0.40	0.37	0.50
11. Camp (L)	777	7	0.64	0.54	0.59	0.40	0.49	0.48	0.44
12. Xin	1,600	8	0.94	0.13	0.39	0.15	0.58	0.29	0.41
13. Baron (M)	1,886	13	0.85	0.20	0.41	0.34	0.42	0.32	0.25
14. Muraro	2,126	10	0.87	0.29	0.46	0.32	0.23	0.36	0.30
15. Segerstolpe	2,209	14	0.88	0.21	0.34	0.35	0.35	0.22	0.24
16. Klein	2,717	4	0.97	0.36	0.46	0.58	0.61	0.54	0.33
17. Romanov	2,881	7	0.69	0.17	0.31	0.29	0.31	0.23	0.28
18. Zeisel	3,005	9	0.73	0.24	0.41	0.41	0.37	0.23	0.30
19. Lake	3,042	16	0.52	0.28	0.37	0.27	0.39	0.32	0.29
20. Puram	5,902	10	0.58	0.08	0.25	0.66	0.65	0.18	0.36
21. Montoro	7,193	7	0.80	0.11	0.23	0.13	0.29	0.19	0.43
22. Baron (H)	8,569	14	0.89	0.10	0.46	0.29	0.65	0.37	0.32
23. Chen	12,089	46	0.68	0.11	0.51	0.49	0.29	0.50	0.23
24. Sanderson	12,648	11	0.89	0.07	0.13	0.11	0.50	0.10	0.18
25. Slyper	13,316	8	0.77	0.07	0.23	0.02	0.62	0.24	0.39
26. Campbell	21,086	21	0.62	0.06	0.30	0.17	0.35	0.17	0.16
27. Zilionis	34,558	9	0.79	0.08	0.27	0.04	0.50	0.29	0.41
28. Macosko	44,808	12	0.76	0.08	0.22	0.50	0.24	0.22	0.28
29. Hrvatin	48,266	8	0.85	0.19	0.34	NA	NA	0.44	0.79
30. Tabula Muris	54,439	40	0.59	0.20	0.40	NA	NA	0.36	0.31
31. Karagiannis	72,914	12	0.51	0.21	0.33	NA	NA	0.29	0.33
32. Orozco	100,055	11	0.75	NA	NA	NA	NA	0.20	0.40
33. Darrah	162,490	14	0.79	NA	NA	NA	NA	0.18	0.19
34. Kozareva	611,034	18	0.99	NA	NA	NA	NA	0.18	NA
Mean Jaccard Index			0.77	0.30	0.40	0.37	0.48	0.34	0.39

Supplementary Table 5. Running time of scDHA, SC3, SEURAT, SINCERA, CIDR, SCANPY, and k-means on 34 single-cell datasets. Overall, scDHA is the fastest and was able to analyze 611,034 cells within 24 minutes.

Dataset	Size	scDHA	SC3	SEURAT	SINCERA	CIDR	SCANPY	k-means
Yan	90	1.24	0.49	1.08	0.03	0.03	0.08	0.03
Goolam	124	1.52	0.46	0.92	0.05	0.04	0.03	0.13
Deng	268	1.51	0.50	0.94	0.05	0.05	0.03	0.37
Pollen	301	1.67	0.75	1.68	0.06	0.06	0.03	0.52
Patel	430	1.24	1.09	1.33	0.02	0.03	0.01	0.19
Wang	457	1.56	0.91	2.18	0.09	0.08	0.03	0.86
Darmanis	466	1.67	0.86	1.32	0.09	0.09	0.04	0.73
Camp (B)	553	1.57	1.10	2.04	0.11	0.08	0.03	0.80
Usoskin	622	1.67	1.44	1.97	0.17	0.17	0.03	1.09
Kolodziejczyk	704	1.89	1.71	2.59	0.29	0.23	0.05	1.79
Camp (L)	777	1.91	1.94	1.90	0.17	0.17	0.03	0.88
Xin	1,600	2.42	12.69	3.43	1.43	0.65	0.08	3.58
Baron (M)	1,886	2.33	15.01	1.43	0.71	0.54	0.04	1.20
Muraro	2,126	2.53	4.27	1.44	1.20	0.77	0.06	1.44
Segerstolpe	2,209	2.54	4.62	3.02	1.77	1.15	0.07	4.23
Klein	2,717	2.54	10.34	4.56	2.26	1.85	0.10	4.28
Romanov	2,881	2.56	8.78	3.08	2.57	2.09	0.07	3.05
Zeisel	3,005	2.50	9.00	3.04	2.51	1.96	0.08	1.90
Lake	3,042	2.53	10.44	4.94	3.11	2.85	0.10	5.78
Puram	5,902	2.72	66.35	3.69	9.39	10.16	0.18	3.62
Montoro	7,193	2.54	59.85	5.42	29.26	18.99	0.14	15.01
Baron (H)	8,569	2.81	55.79	3.36	28.93	30.73	0.37	6.49
Chen	12,089	3.00	67.84	8.51	53.33	73.57	0.24	22.98
Sanderson	12,648	2.57	59.44	3.96	33.39	74.31	0.31	7.20
Slyper	13,316	2.92	53.89	3.91	50.44	96.38	0.90	17.50
Campbell	21,086	3.60	77.56	11.19	164.04	372.83	0.56	34.05
Zilionis	34,558	4.68	87.73	29.85	764.05	2146.26	1.24	61.36
Macosko	44,808	4.49	96.58	26.40	614.65	3312.65	1.52	86.58
Hrvatin	48,266	4.81	86.76	19.11	NA	NA	1.39	40.00
Tabula Muris	54,439	11.52	89.16	19.23	NA	NA	2.21	66.90
Karagiannis	72,914	12.58	60.29	41.47	NA	NA	1.97	97.86
Orozco	100,055	11.80	NA	NA	NA	NA	12.06	189.99
Darrah	162,490	14.63	NA	NA	NA	NA	14.70	264.57
Kozareva	611,034	23.90	NA	NA	NA	NA	35.45	NA

1.4 Simulation study

We compared the clustering performance using simulation studies with 25 simulated datasets. To simulate the data, we used Splatter R package[7] which has been utilized for simulation in recent research articles[8,9,10] and surveys[11,12]. This software allows us to generate data with different sizes and sparsity levels. Using Splatter, we generated 25 simulated datasets with varying number of cells and sparsity levels. We set the number of genes to 10,000 and generated datasets with different number of cells: 5,000, 10,000, 25,000, 50,000, and 100,000. For each of the five settings, we simulated 5 sparsity levels by adjusting the *dropout.mid* parameters (midpoint parameter for dropout logistic function of Splatter). We set *dropout.mid* to 0, 0.5, 1, 1.5, and 2, which led to sparsity levels of 28%, 32%, 37%, 44%, 51%, respectively. In each dataset, we simulated five cell types. The true cell labels were used to assess the performance of the clustering methods.

Supplementary Figure 1a shows the running time of each method. The horizontal axis shows the varying number of cells while the vertical axis shows the running time (in minutes). SINCERA and CIDR crashed when analyzing datasets with 50,000 cells or more. Similar to the analysis of real datasets, scDHA and SCANPY are the fastest among the compared methods. They were able to analyze 100,000 cells in minutes. Supplementary Figures 1b–f show the ARI values obtained from comparing the discovered groups against thee ground truth. Overall, scDHA has the highest ARI values in our analysis.

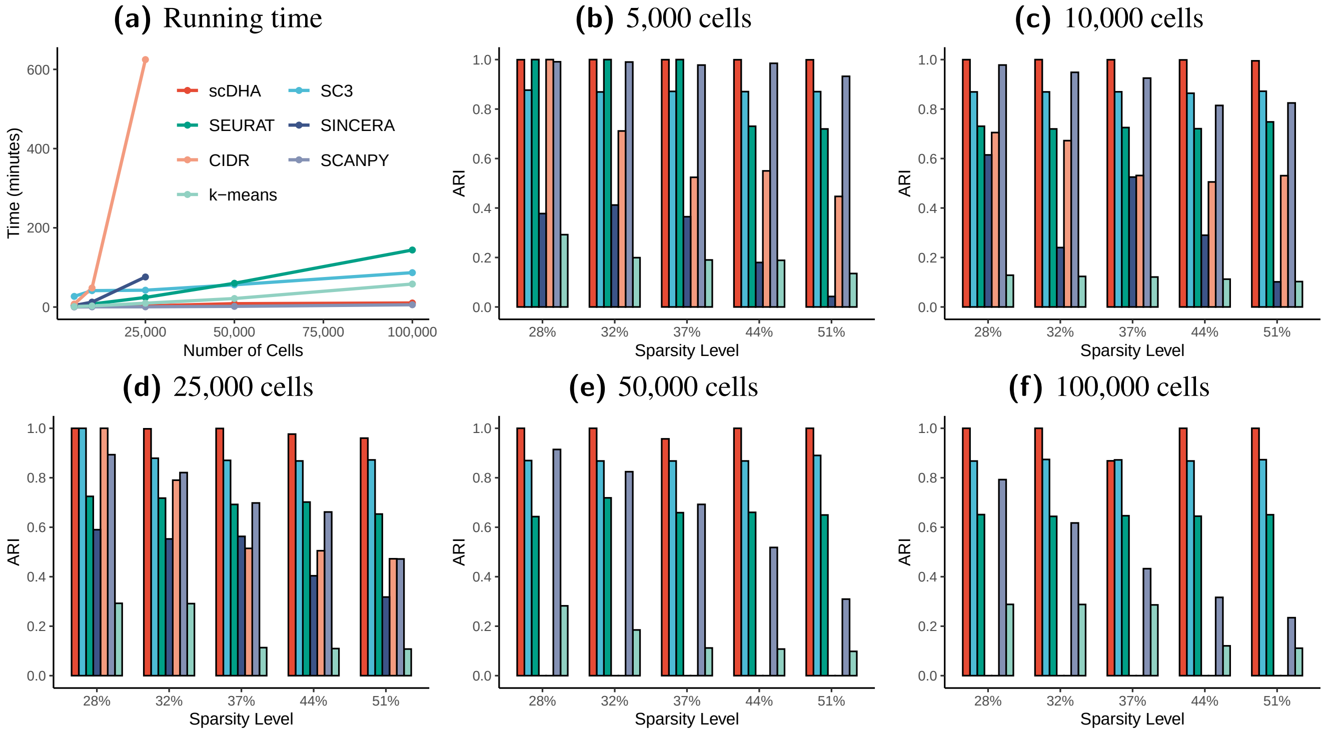

Supplementary Figure 1. Analysis results using 25 simulated datasets. (a) Running time of the clustering methods, each using 10 cores. scDHA and SCANPY are the fastest among the seven methods. (b)-(f) Clustering performance of scDHA, SC3, SEURAT, SINCERA, CIDR, SCANPY, and k-means on simulated data with 5,000, 10,000, 25,000, 50,000, and 100,000 cells, respectively. The x-axis shows sparsity levels. Accuracy is measured by adjusted Rand Index (ARI). scDHA has the highest average ARI across 25 simulated datasets.

1.5 Impact of scRNA-seq normalization

In our analysis, single-cell datasets were processed in various ways by the data providers: 12 datasets had raw counts, 6 datasets had CPM-normalized data, 8 datasets were RPKM-normalized, and 8 datasets were TPM-normalized. To better understand the effect of normalization on scDHA performance, we have re-analyzed the datasets using different normalization techniques: TPM, CPM, and RPKM. We were able to convert most datasets to TPM, CPM, and RPKM. We were not able to convert the data from UMI values of only two datasets: Darrah (162,490 cells) and Kozavera (611,034 cells) due to excessive memory usage of the conversion. Supplementary Figure 2 shows the ARI values obtained from cluster analysis using different normalization techniques. Indeed, TMP normalization offers an improvement in performance. Although the difference is marginal, we recommend users to use TPM normalization for a better analysis.

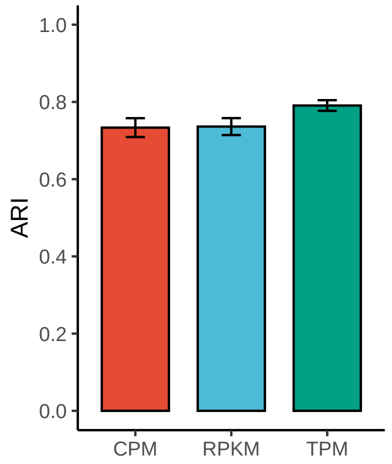

Supplementary Figure 2. Performance of scDHA on 32 single-cell datasets using different normalization metrics: CPM, RPKM, and TPM. Data are presented as mean values +/- variance.

1.6 Impact of min-max scaling on scDHA

To reduce the technical variability and heterogeneous calibration from sequencing technologies, scDHA rescales the expression data to range from 0 to 1 for each cell. The 0-1 scaling is not a scRNA-seq normalization method and it is not intended to do so. This min-max scaling added to our method is used on top of the already normalized data provided by users. Such scaling is frequently used in deep learning models[13,14,15,16] with the common purpose of reducing standard deviation and suppressing the effect of outliers. To demonstrate the impact of this min-max scaling, we re-analyzed the 34 single-cell datasets using scDHA without scaling the data to 0-1. Supplementary Figure 3 shows the ARI values obtained from scDHA in two different scenarios: scDHA with and without the scaling step. Overall, the 0-1 scaling makes the analysis more robust (lower variance) and more accurate (higher ARI).

1.7 Impact of non-negative constraint on scDHA

We observe that allowing negative weights will lead to overfitting and potentially the selection of less important genes. To demonstrate the importance of this constraint, we analyzed the data without enforcing the weight to be non-negative in the first module. Supplementary Figure 4 shows the ARI values obtained from scDHA in two different scenarios: scDHA with and without the non-negative constraint. The ARI values obtained from scDHA without non-negative constrain has higher variability and lower ARI values.

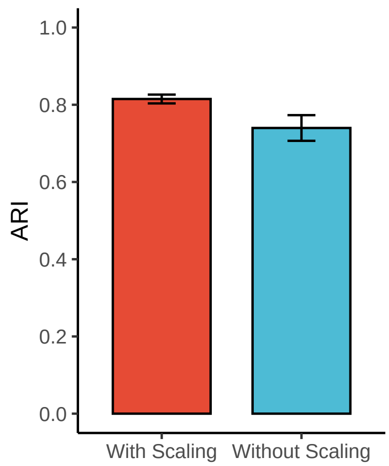

Supplementary Figure 3. Impact of min-max scaling on scDHA clustering performance on 34 single-cell datasets. Data are presented as mean values +/- variance. The min-max scaling provides more stability (lower variance) and higher accuracy (higher ARI).

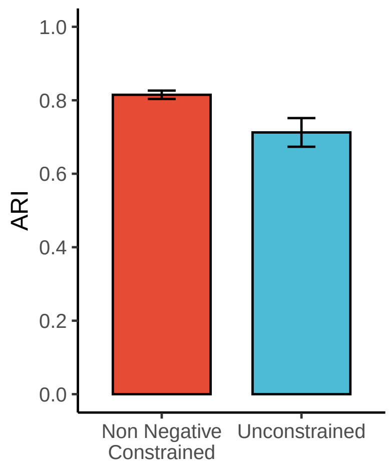

Supplementary Figure 4. Impact of non-negative constraint on scDHA clustering performance on 34 single-cell datasets. Data are presented as mean values +/- variance. The constraint provides more stability (lower variance) and higher accuracy (higher ARI).

1.8 Impact of model parameters on scDHA

In the denoising autoencoder, the bottleneck layer is set to a fixed size of 50 nodes. We have tested the software with different numbers of nodes and found that varying this number does not have a significant impact on the performance of the software. As shown in Supplementary Figure 5, the average ARI value of the clustering results is consistently at 0.8 when we vary the number of nodes from 30 to 70.

Based on the computed weights of denoising module, we choose 5,000 genes with the highest weight variances (also the default setting). Supplementary Figure 6a shows the normalized weight variances in which each line represents a dataset. The figure shows that most lines are flattened at 5,000 genes. Another important note is that changing this threshold does not have a significant impact on the overall performance of scDHA. Supplementary Figure 6b shows the clustering performance of scDHA with varying number of genes. The average ARI is consistently close to 0.8 when we change the number of genes from 3,000 to

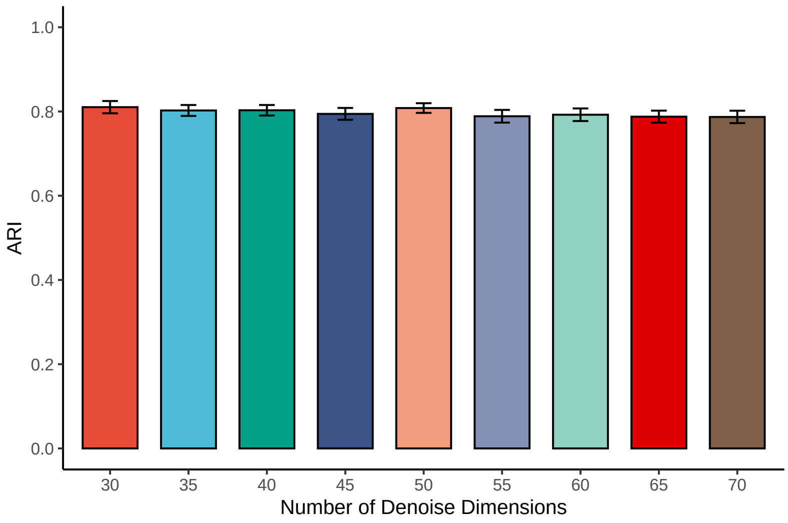

Supplementary Figure 5. Clustering performance of scDHA on 34 single-cell datasets with varying size of bottleneck layer in the first module. Data are presented as mean values +/- variance.

10,000.

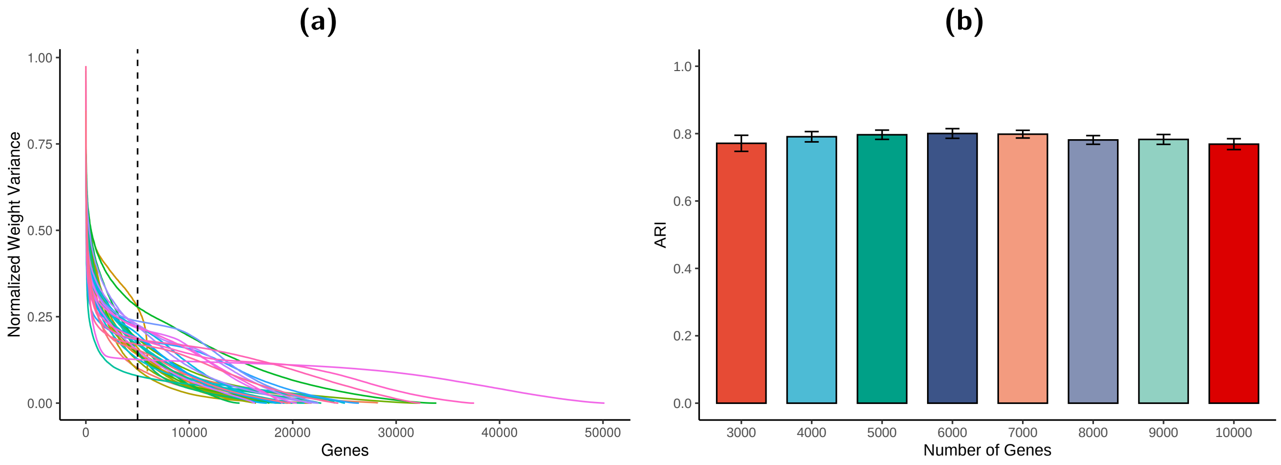

Supplementary Figure 6. Effect of gene filtering cutoff on scDHA performance. (a) Normalized weight variance of genes. (b) Performance of scDHA on 34 single-cell datasets with varying number of selected genes. Data are presented as mean values +/- variance.

1.9 Predicting the number of cell types for Klein dataset

The transcriptome landscape of the Klein dataset is shown in Supplementary Figure 7. This dataset consists of four cell types: d0, d2, d4, and d7. As shown in the landscape, d0 and d7 are well-separated from the rest of the cells. The remaining classes, d2 and d4, are separable but they are closer to one another than to the rest.

The number of cell types is determined using two indices: (i) the ratio of *between sum of squares* over the *total sum of squares*, and (ii) the increase of the *within sum of squares* when number of cluster

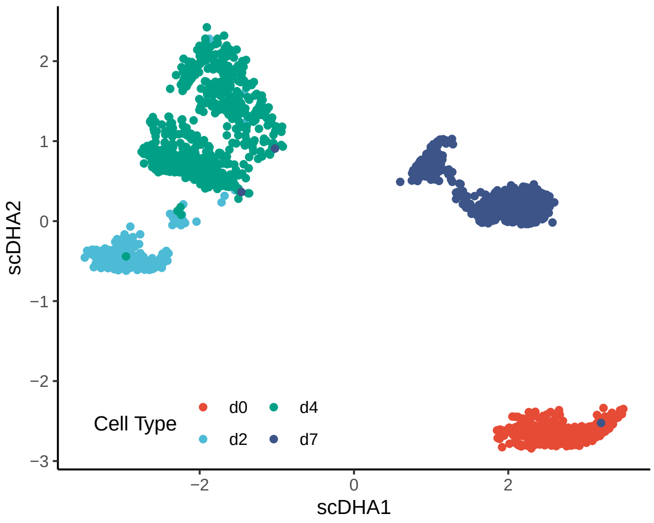

Supplementary Figure 7. The 2D visualization of the Klein dataset using scDHA. The data consists of four cell types: d0,d2, d4, and d7.

increases. Denoting j as the number of clusters, the indices are formulated as follows:

$$Index\ 1 = \frac{SS_{between,j}}{SS_{total,j}}$$

$$Index\ 2 = \frac{SS_{within,j+1} - SS_{within,j}}{SS_{within,j}}$$

Supplementary Figure 8a shows the values of SS_{total}, $SS_{between}$, and SS_{within} while Supplementary Figure 8b shows the values of *Index 1* and *Index 2*. *Index 1* is the ratio of $SS_{between}$ to SS_{total}, which reflects the relative distance among the clusters. By maximizing *Index 1*, we aim at maximizing the relative distance between the discovered clusters. This index peaks at $j = 4$, which is the true number of cell types. However, since the two classes d2 and d4 are close to one another, *Index 1* has very similar values for $j = 3$ and $j = 4$.

On the other hand, *Index 2* quantifes the relative increase of SS_{within} for the *next value* of j. By maximizing this index, we find an optimal j for which $j+1$ has the largest relative increase of SS_{within} (which is undesirable). As shown in Supplementary Figure 8a, the SS_{within} decreases when $j \in [2\dots4]$ but increases when j is higher than 4. This index clearly indicates that we should stop at $j = 4$ because $j = 5$ would lead to an increase of SS_{within}.

Note that the two indices have similar shapes (with different scales). When *Index 1* increases, *Index 2* is likely to increase and vice versa. However, the denominator of *Index 2* is SS_{within}, which is much smaller than SS_{total}. This makes *Index 2* sensitive to even a small change of SS_{within}. By default, scDHA sets the mean of the two indices to be the optimal number of clusters.

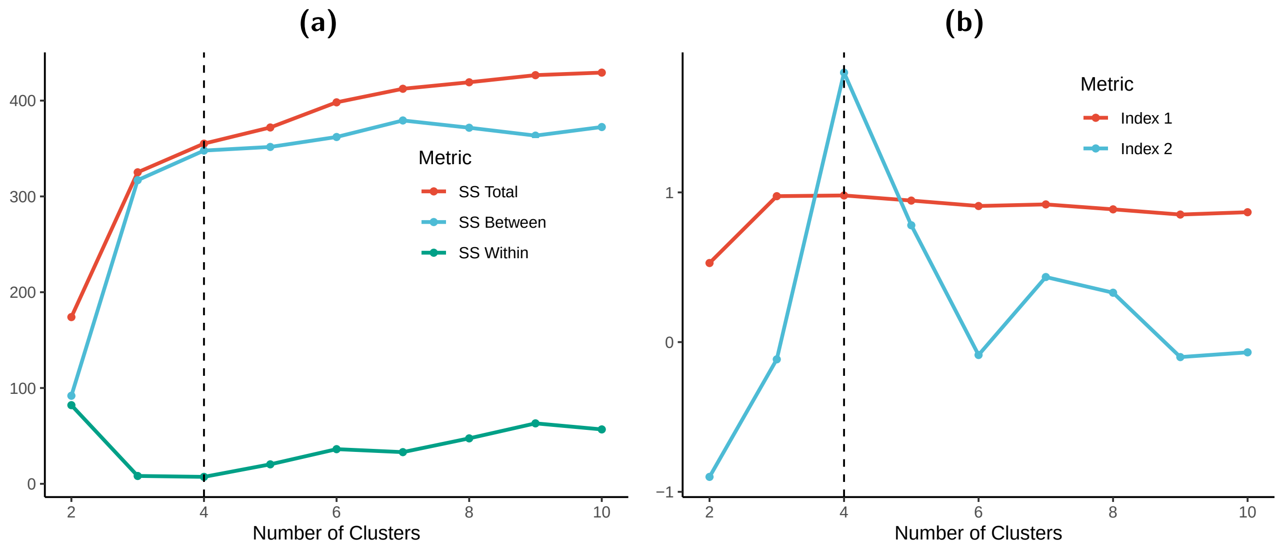

Supplementary Figure 8. Metrics for determining the number of clusters, demonstrated on the Klein dataset. (a) SS_{total} (red), $SS_{between}$ (blue), and SS_{within} (green). (a) *Index 1* (red) and *Index 2* (blue). Both indices indicate the optimal number of clusters is 4.

2 Supplementary Note 2: Dimension reduction and visualization

2.1 Performance evaluation

We compare scDHA with PCA, t-SNE[17,18], UMAP[19,20], and SCANPY. Supplementary Table 6 shows the silhouette index of the 34 dataset using each method while Supplementary Table 7 shows the running time. For the Kozareva dataset, only scDHA and SCANPY can generate its 2D representation. Moreover, t-SNE produces errors when the data matrix is too big. Therefore, for two big datasets, Orozco and Darrah, we perform PCA on the original data and input the first 50 PCs into t-SNE function (same as t-SNE default setting). The color-coded representations are shown in Supplementary Figures 9–17. In each representation, we calculate the silhouette index that measures the cohesion among the cells of the same type and the separation between different cell types. scDHA offers a significant improvement over current state-of-the-art techniques.

Supplementary Table 6. Silhouette values calculated for representation using scDHA, PCA, t-SNE, UMAP, and SCANPY. Cells with NA values indicate that the method was not able to analyze the dataset (out-of-memory). Cells highlighted in green have the highest silhouette values. scDHA has the highest average silhouette value. It outperforms other methods in 25 out of 34 datasets.

Dataset	Size	scDHA	PCA	t-SNE	UMAP	SCANPY
Yan	90	0.52	0.54	0.45	0.47	0.73
Goolam	124	0.37	0.31	0.28	0.27	-0.02
Deng	268	0.50	0.60	0.49	0.67	0.45
Pollen	301	0.78	0.30	0.61	0.58	0.65
Patel	430	0.62	0.17	0.52	0.52	0.31
Wang	457	0.28	-0.07	0.13	0.21	0.27
Darmanis	466	0.47	0.01	0.31	0.34	0.25
Camp (B)	553	0.54	0.07	0.36	0.30	0.34
Usoskin	622	0.62	0.07	0.40	0.51	0.45
Kolodziejczyk	704	0.81	0.30	0.43	0.54	0.50
Camp (L)	777	0.67	0.17	0.42	0.50	0.41
Xin	1,600	0.67	0.08	0.25	0.17	0.36
Baron (M)	1,886	0.44	-0.23	0.05	0.10	0.43
Muraro	2,126	0.57	-0.20	0.24	0.46	0.24
Segerstolpe	2,209	0.66	-0.22	0.01	0.24	0.22
Klein	2,717	0.72	0.24	0.48	0.69	0.69
Romanov	2,881	0.37	0.03	0.24	0.34	0.27
Zeisel	3,005	0.67	0.03	0.31	0.55	0.34
Lake	3,042	0.35	-0.11	0.25	0.32	0.29
Puram	5,902	0.27	0.23	0.05	0.28	0.24
Montoro	7,193	0.16	0.24	0.09	0.29	0.22
Baron (H)	8,569	0.61	-0.14	0.20	0.46	0.50
Chen	12,089	0.49	-0.07	0.09	0.35	0.40
Sanderson	12,648	0.09	0.06	0.04	0.16	0.14
Slyper	13,316	0.44	0.22	0.16	0.44	0.45
Campbell	21,086	0.01	-0.31	-0.05	-0.08	0.03
Zilionis	34,558	0.44	0.00	0.22	0.42	0.29
Macosko	44,808	0.27	0.11	0.09	0.36	0.27
Hrvatin	48,266	0.73	0.36	0.26	0.59	0.46
Tabula Muris	54,439	0.11	-0.24	-0.14	-0.07	-0.17
Karagiannis	72,914	0.05	-0.08	0.05	0.17	0.11
Orozco	100,055	0.72	0.69	-0.15	0.02	0.22
Darrah	162,490	0.26	-0.36	-0.15	0.17	0.13
Kozareva	611,034	0.76	NA	NA	NA	0.50
Mean		0.47	0.08	0.21	0.33	0.32

Supplementary Table 7. Running time of scDHA, PCA, t-SNE, UMAP, and SCANPY on 34 single cell datasets. Cells with NA values indicate that the method was not able to analyze the dataset (out-of-memory).

Dataset	Size	scDHA	PCA	t-SNE	UMAP	SCANPY
Yan	90	1.42	0.00	0.02	0.00	0.11
Goolam	124	1.70	0.01	0.10	0.17	0.05
Deng	268	1.71	0.01	0.09	0.39	0.05
Pollen	301	1.81	0.02	0.13	0.41	0.05
Patel	430	1.43	0.02	0.07	0.17	0.04
Wang	457	1.78	0.02	0.20	0.67	0.06
Darmanis	466	1.83	0.02	0.21	0.67	0.07
Camp (B)	553	1.79	0.02	0.25	0.67	0.06
Usoskin	622	1.86	0.02	0.33	1.09	0.07
Kolodziejczyk	704	2.06	0.05	0.56	1.82	0.10
Camp (L)	777	2.08	0.02	0.14	0.67	0.07
Xin	1,600	2.68	0.10	0.98	2.90	0.17
Baron (M)	1,886	2.70	0.04	0.29	0.98	0.15
Muraro	2,126	2.92	0.07	0.31	1.36	0.17
Segerstolpe	2,209	2.89	0.09	0.72	1.98	0.18
Klein	2,717	2.89	0.10	1.40	3.48	0.24
Romanov	2,881	2.90	0.10	0.53	2.68	0.22
Zeisel	3,005	2.91	0.08	0.37	1.43	0.23
Lake	3,042	2.96	0.11	0.67	3.38	0.27
Puram	5,902	3.59	0.22	0.83	3.32	0.52
Montoro	7,193	4.25	0.29	2.65	6.38	0.52
Baron (H)	8,569	5.34	0.29	1.18	3.33	0.83
Chen	12,089	6.03	0.47	1.99	7.18	0.51
Sanderson	12,648	4.48	0.43	1.83	3.97	0.57
Slyper	13,316	4.53	0.60	7.56	16.72	1.26
Campbell	21,086	6.85	1.07	4.18	10.93	1.02
Zilionis	34,558	11.55	3.72	21.06	59.90	1.97
Macosko	44,808	7.82	2.09	12.49	24.11	2.51
Hrvatin	48,266	8.01	2.27	13.26	51.68	2.45
Tabula Muris	54,439	11.10	1.69	7.19	13.89	3.54
Karagiannis	72,914	13.26	3.33	22.83	73.35	3.54
Orozco	100,055	21.74	12.48	56.70	145.85	14.61
Darrah	162,490	31.92	10.54	42.78	92.54	18.53
Kozareva	611,034	62.11	NA	NA	NA	53.02

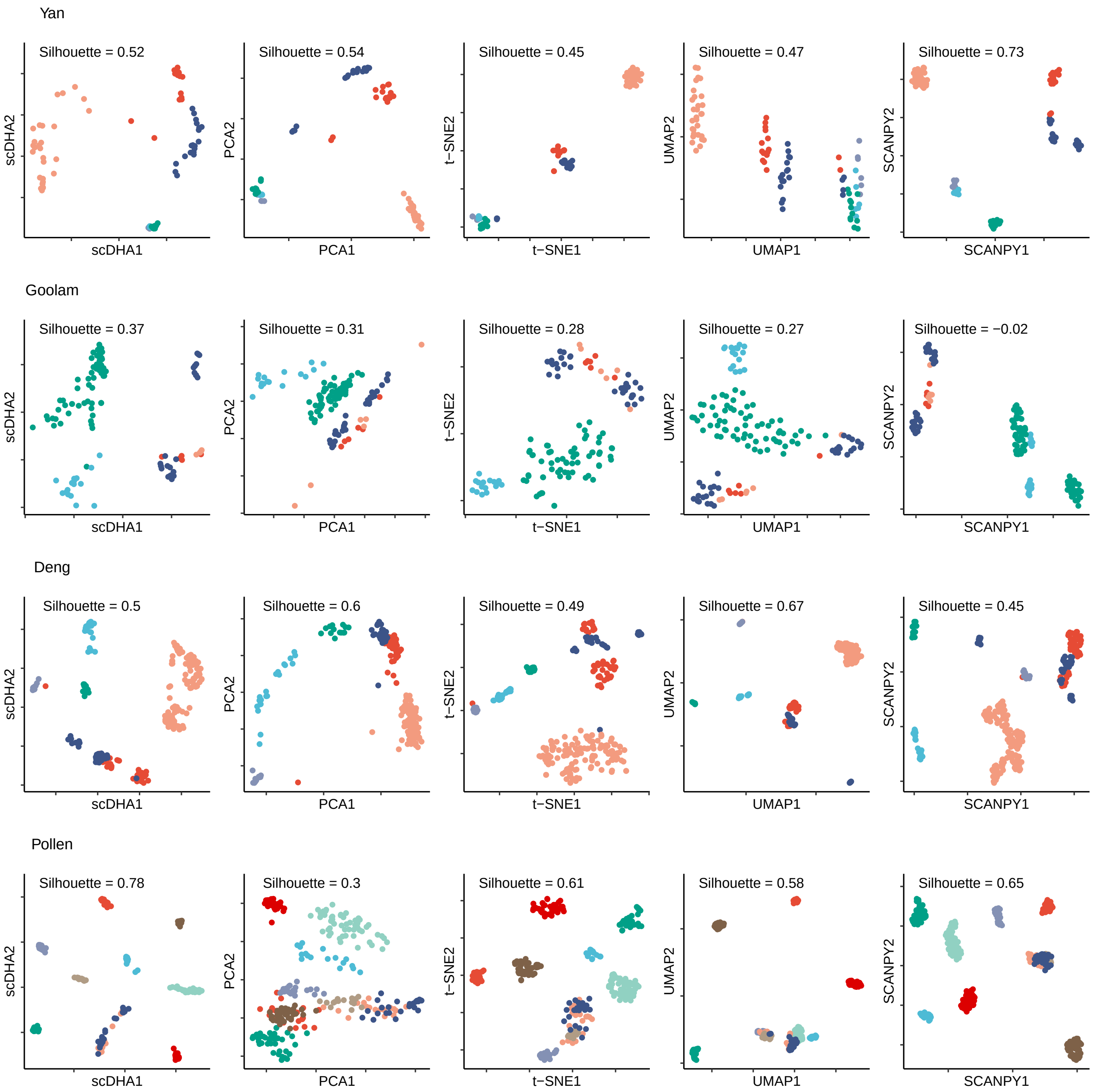

Supplementary Figure 9. Representation of the Yan, Gollam, Deng, and Pollen datasets (top to bottom) using scDHA, PCA, t-SNE, UMAP, and SCANPY (left to right). Different colors code for different cell types.

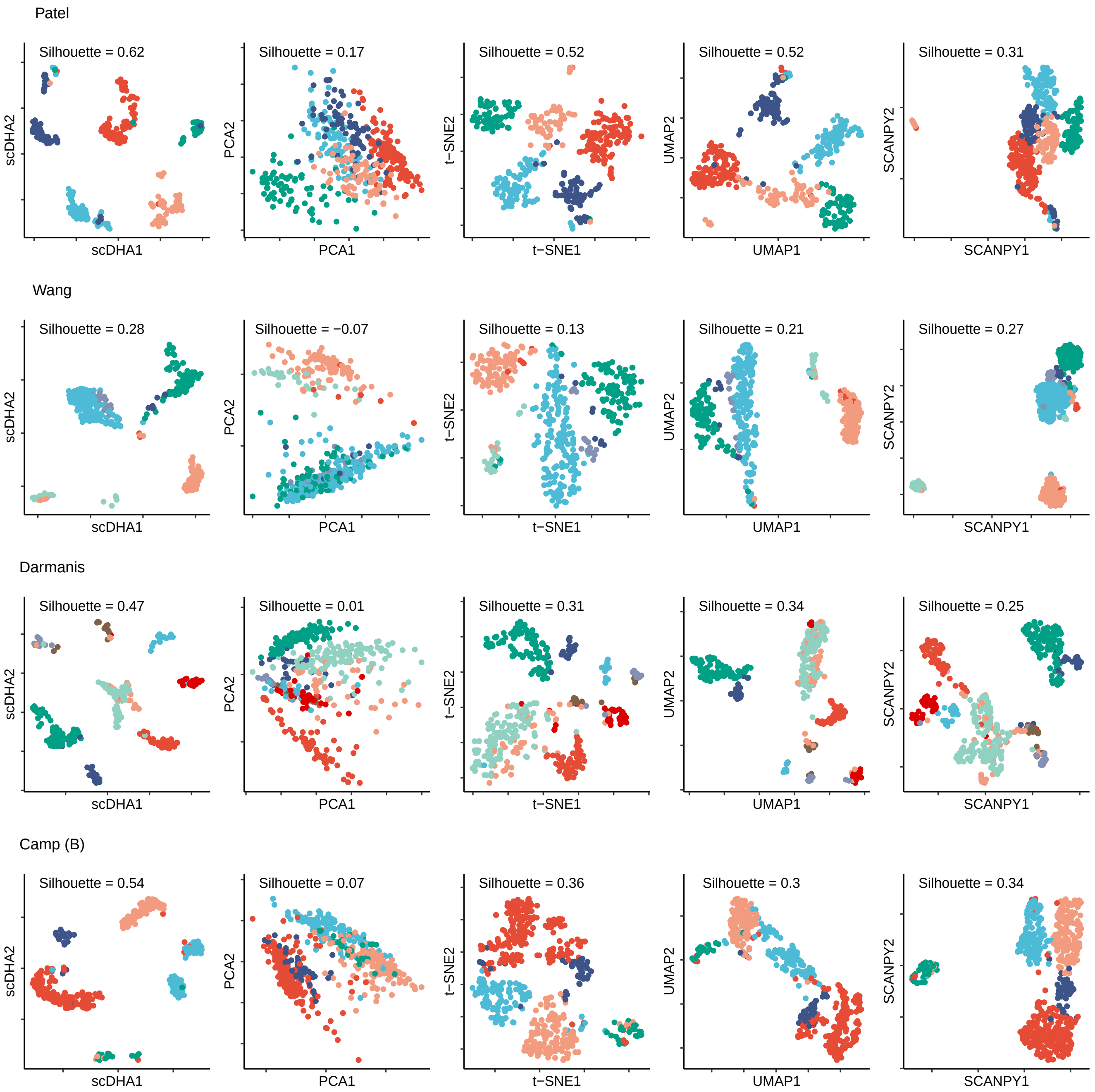

Supplementary Figure 10. Representation of the Patel, Wang, Darmanis, and Camp (Brain) datasets (top to bottom) using scDHA, PCA, t-SNE, UMAP, and SCANPY (left to right). Different colors code for different cell types.

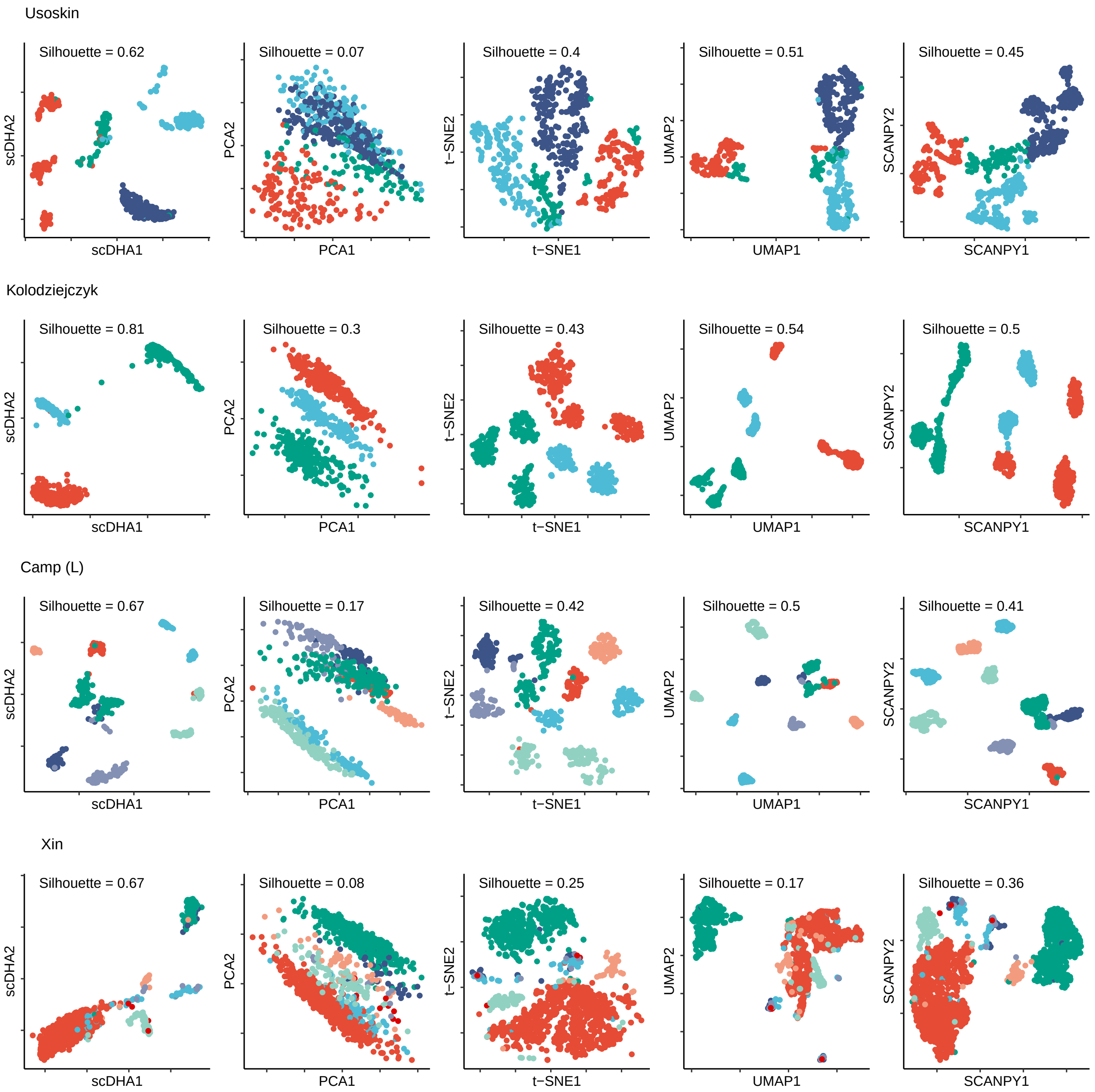

Supplementary Figure 11. Representation of Usoskin, Kolodziejczyk, Camp (Liver), and Xin datasets (top to bottom) using scDHA, PCA, t-SNE, UMAP, and SCANPY (left to right). Different colors code for different cell types.

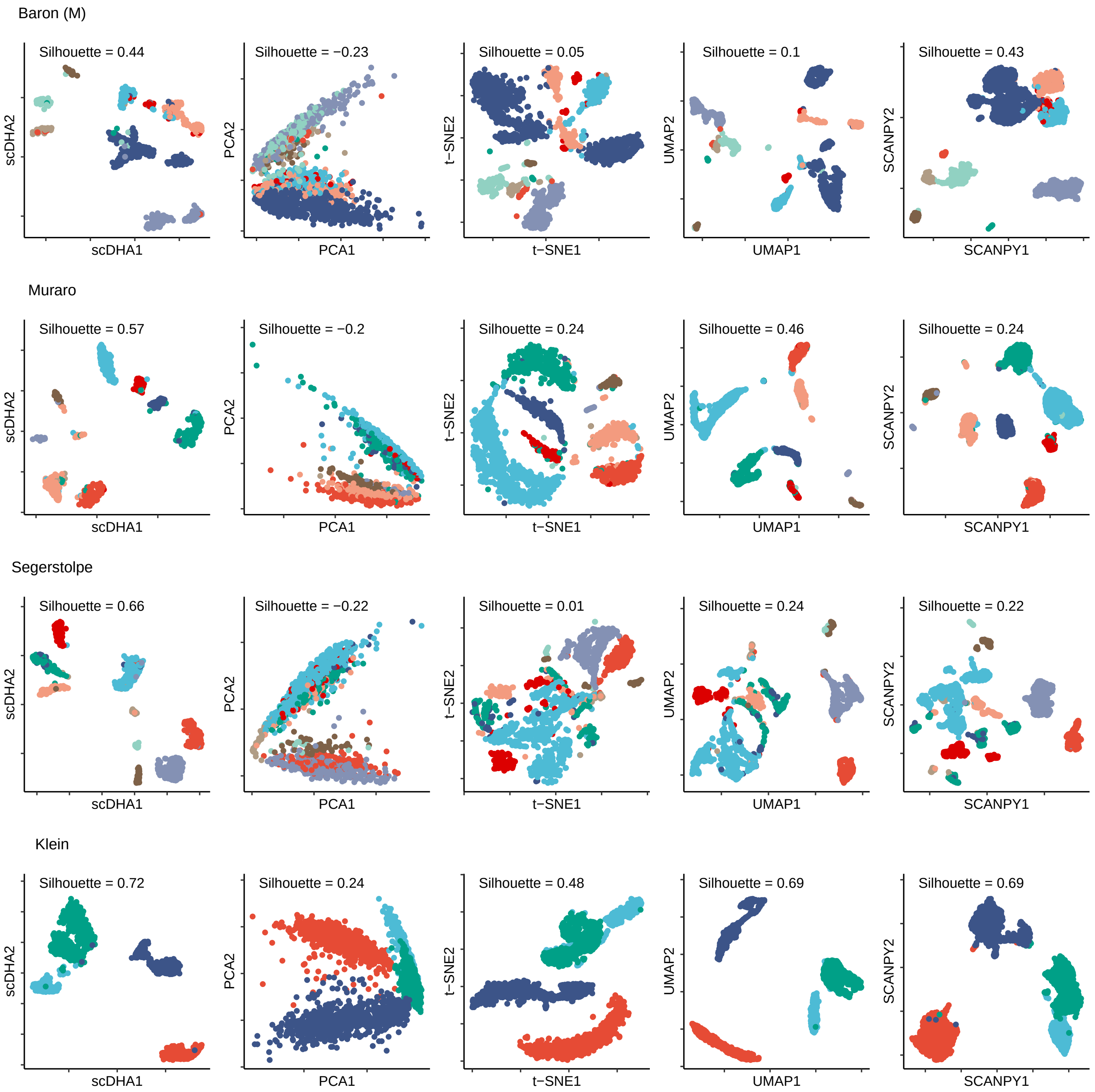

Supplementary Figure 12. Representation of Baron (mouse), Muraro, Segerstolpe, and Klein datasets (top to bottom) using scDHA, PCA, t-SNE, UMAP, and SCANPY (left to right). Different colors code for different cell types.

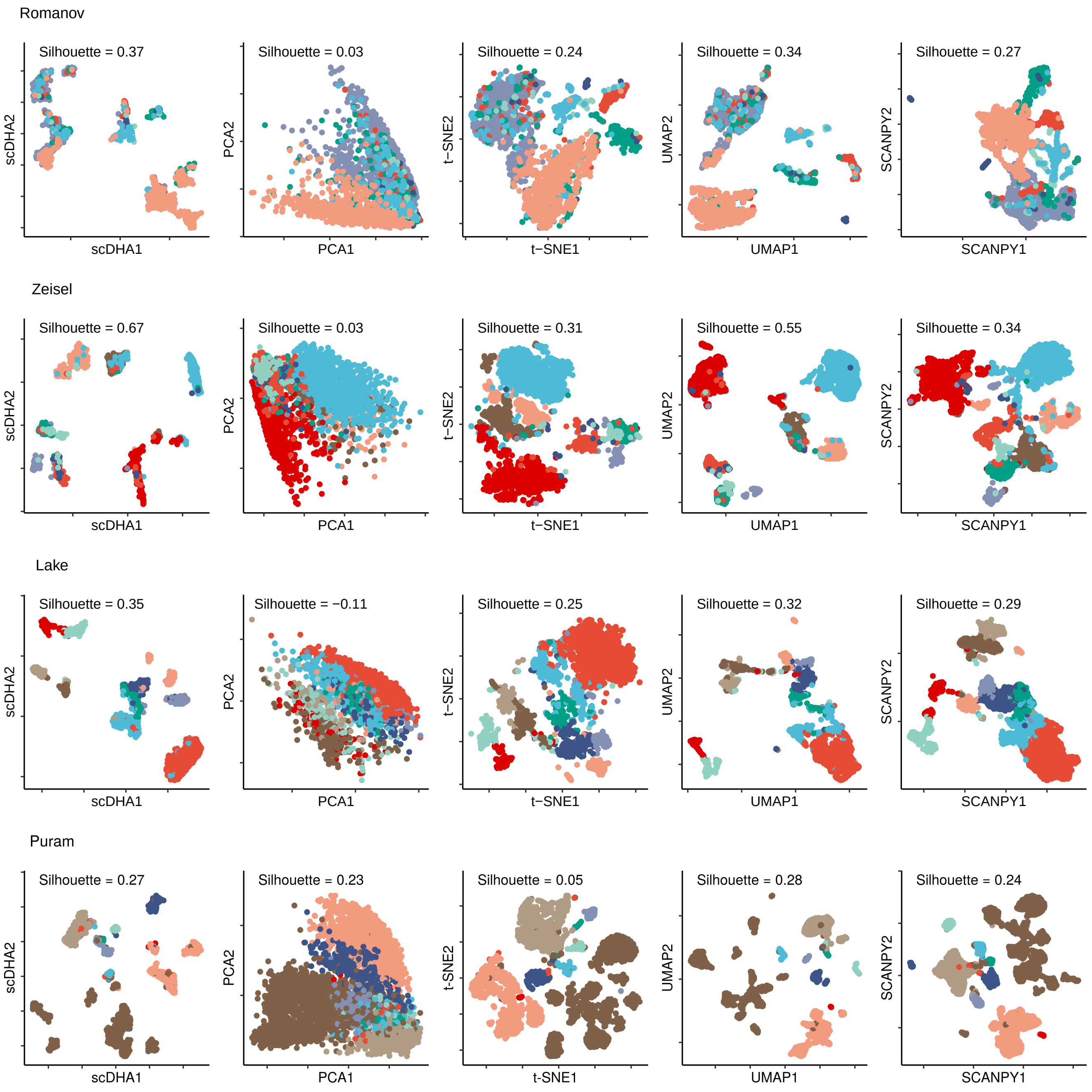

Supplementary Figure 13. Representation of Romanov, Zeisel, Lake, and Puram datasets (top to bottom) using scDHA, PCA, t-SNE, UMAP, and SCANPY (left to right). Different colors code for different cell types.

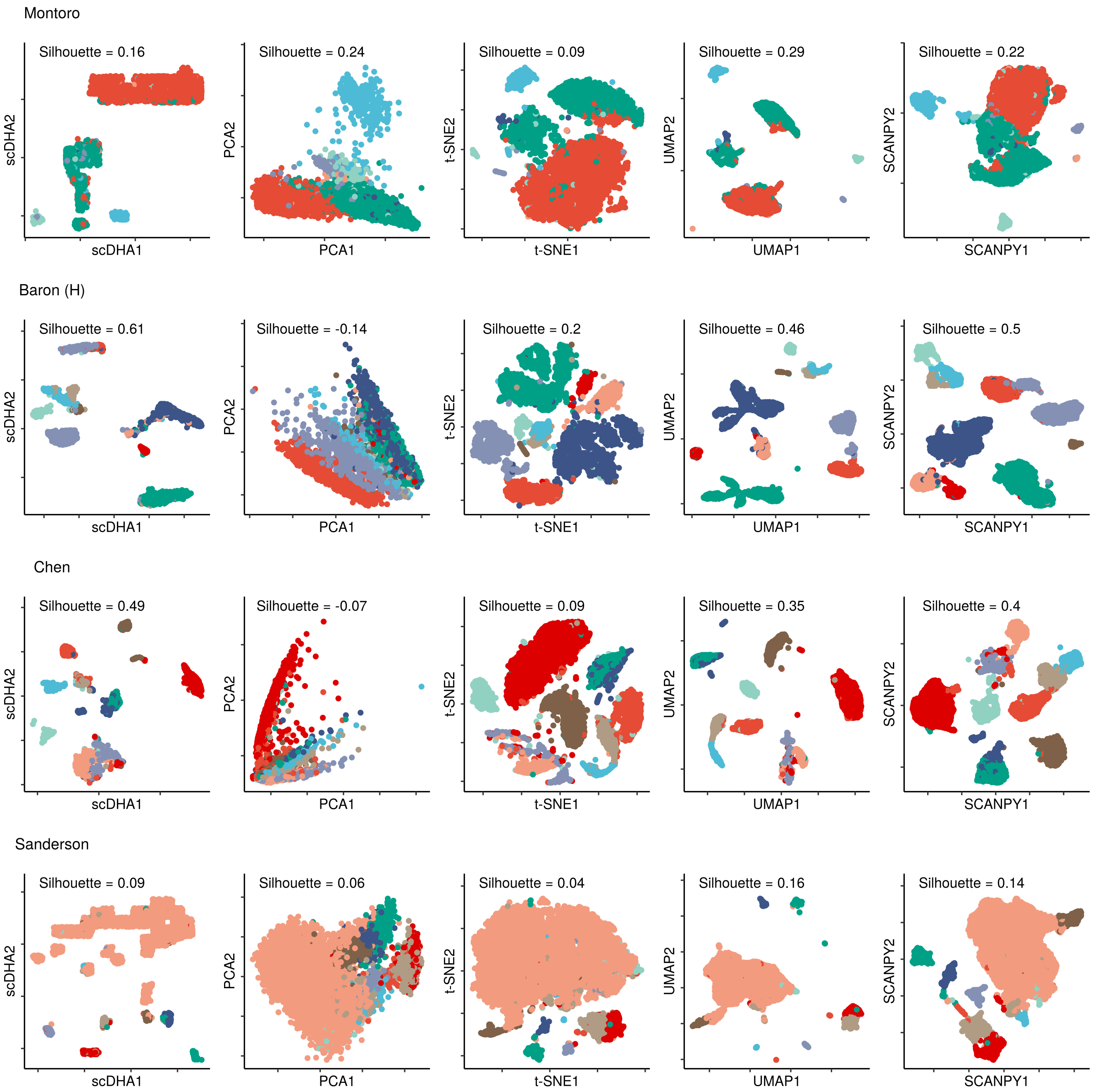

Supplementary Figure 14. Representation of Montoro, Baron (Human), Chen, and Sanderson datasets (top to bottom) using scDHA, PCA, t-SNE, UMAP, and SCANPY (left to right). Different colors code for different cell types.

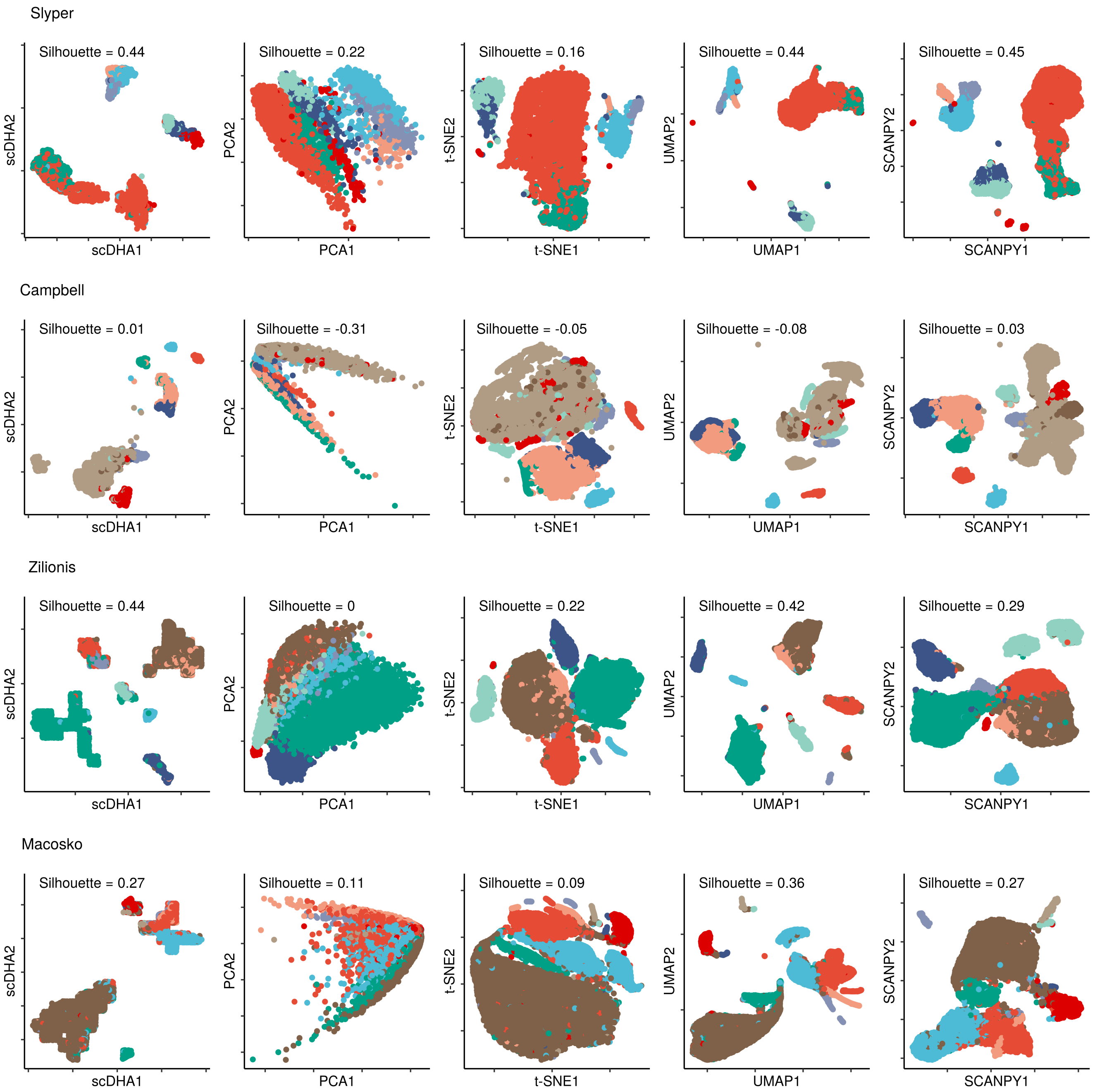

Supplementary Figure 15. Representation of Slyper, Campbell, Zilionis, and Macosko datasets (top to bottom) using scDHA, PCA, t-SNE, UMAP, and SCANPY (left to right). Different colors code for different cell types.

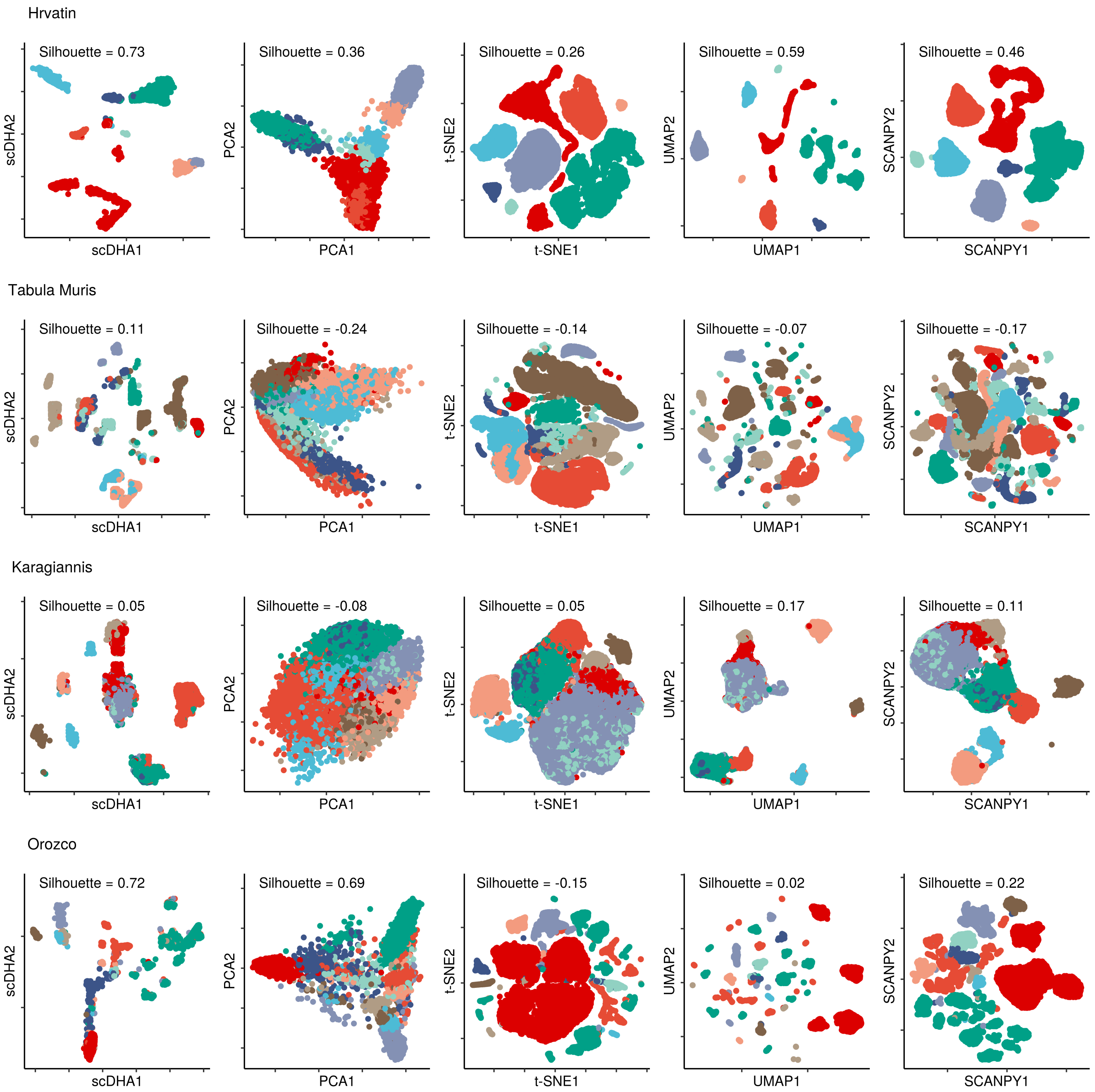

Supplementary Figure 16. Representation of Hrvatin, Tabula Muris, Karagiannis, and Orozco datasets (top to bottom) using scDHA, PCA, t-SNE, UMAP, and SCANPY (left to right). Different colors code for different cell types.

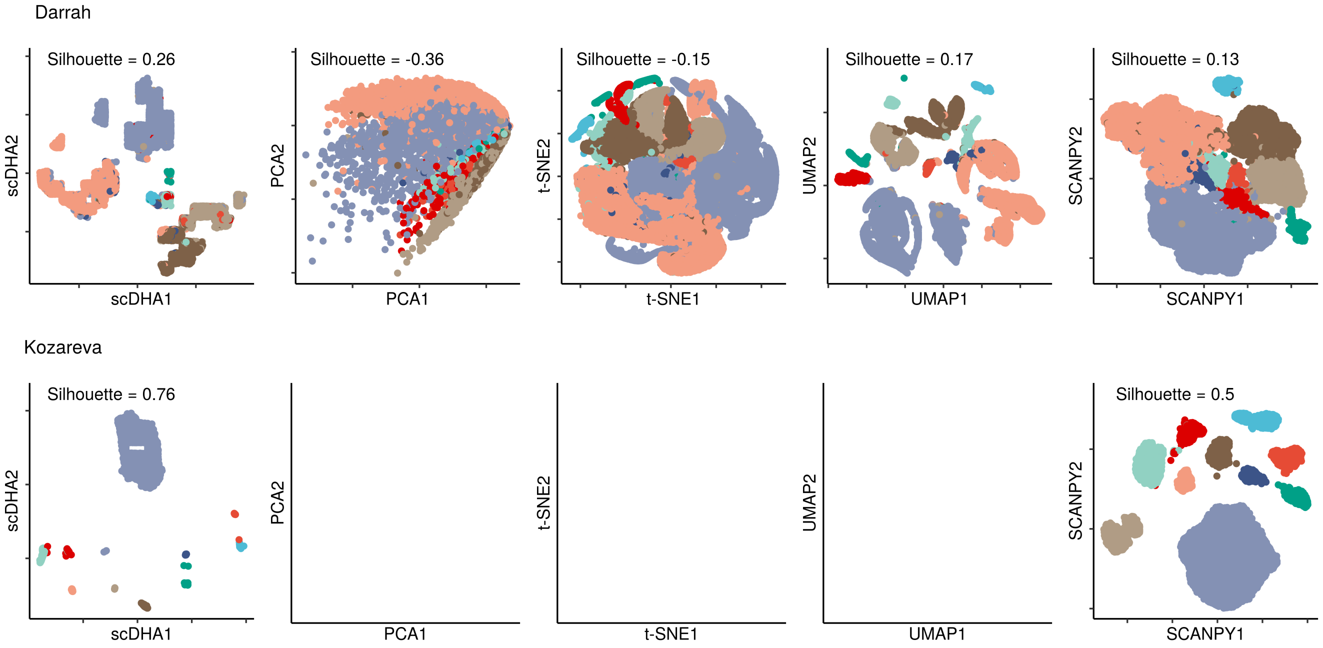

Supplementary Figure 17. Representation of Darrah, and Kozareva datasets (top to bottom) using scDHA, PCA, t-SNE, UMAP, and SCANPY (left to right). Different colors code for different cell types. For Kozareva dataset, only scDHA and SCANPY can generate the 2D representation.

2.2 Batch effects in the Kolodziejczyk dataset

The global structure represented by PCA shows no visible batch effects within each cell type (second panel in Figure 1d in main text). The batch effects are visible in the representations of t-SNE, UMAP, and SCANPY, which focus on preserving the local structure. Even though, these methods are able to merge some batches together. The *2i cells* (red cells in Figure 1d) consists of four batches (chip1 - 82 cells, chip2 - 59 cells, chip3 - 72 cells, and chip4 - 82 cells). Both t-SNE and UMAP are able to merge them into two cell groups: chip2, chip3, and chip4 were merged together and were separated from chip1. SCANPY also merge the batches for *lif* cells. This indicates that the difference between batches is relatively small compared to the difference between cell types.

To diminish batch effects, we increase the perplexity of t-SNE and the number of neighbors in UMAP. By increasing this parameter, we force the algorithms to focus more on the global structure of the data, thus diminishing the difference among batches (Supplementary Figure 18). However, increasing this parameter also decreases the distance among different cell types in the 2D visualization. Supplementary Figure 18 clear shows that cells of different types are visibly closer to one another when we increase the perplexity from 30 to 200 (or neighbor numbers from 15 to 200). The silhouette values obtained for the setting of 200 are even smaller than the silhouette values obtained using the default parameters (perplexity of 30 and neighbor number of 15 are default values).

In this particular dataset, scDHA was able to overcome batch effects not because of its visualization algorithm, but because of the filtering step implemented in the hierarchical autoencoder. In the first module (non-negative kernel autoencoder), scDHA removes genes with insignificant contributions to the part-based representation. The first module removes genes that are differentially expressed due to batch effects but do not have significant contributions to the global data structure. This step diminishes batch effects without decreasing the distance between different cell types (Supplementary Figure 19).

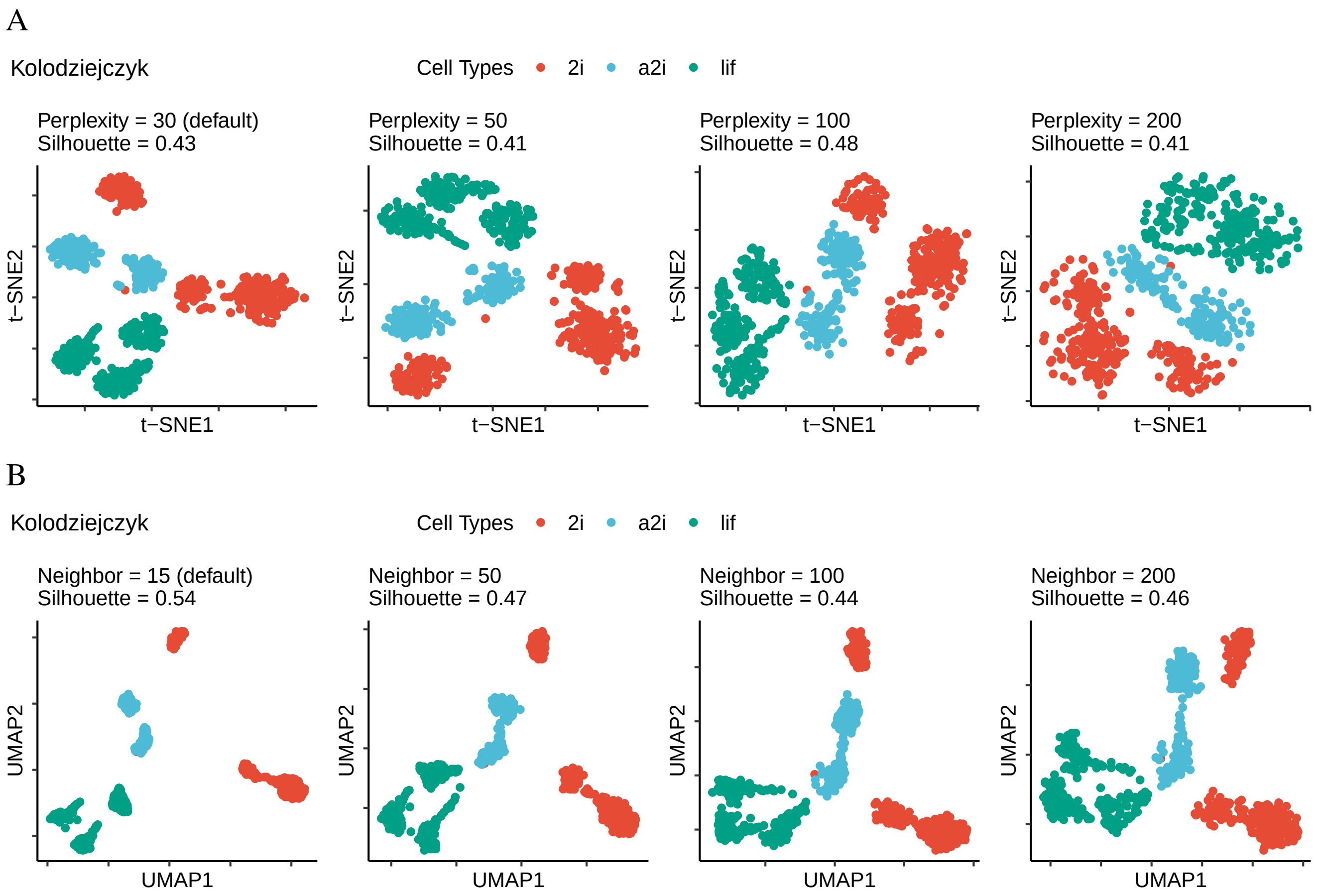

Supplementary Figure 18. Representation of Kolodziejczyk dataset using t-SNE (A) and UMAP (B). The representations are generated with different parameter settings (different perplexity values for t-SNE and different numbers of neighbors for UMAP).

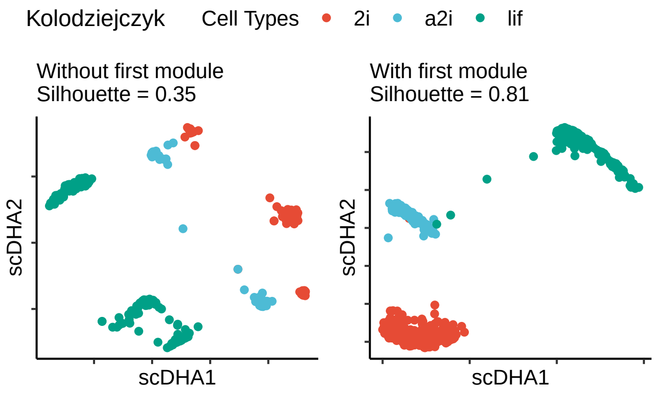

Supplementary Figure 19. Representation of Kolodziejczyk dataset using scDHA with and without the first module. Without the first module, scDHA would be more sensitive to batch effects (left panel). The first module filters out genes that were impacted by batch effects but have insignificant contributions to the global structure, thus increasing the silhouette value from 0.35 to 0.81 (right panel).

3 Supplementary Note 3: Cell classification

In this work, we compare scDHA with XGBoost[21], Random Forest (RF)[22], Deep Learning (DL)[23], and Gradient Boosting Machine (GBM)[24]. We use five datasets related to human pancreas to test the five classification methods. To calculate accuracy metric for classification comparison, we divide number of correct predictions to total number of samples. The accuracy for each evaluation sets is reported in the table below. Moreover, as seen in Supplementary Figure 20, the running of our approach is much faster than other methods in comparison.

Supplementary Table 8. Classification performance measuring by accuracy of scDHA, XGBoost, Random Forest (RF), Deep Learning (DL), and Gradient Boosting Machine (GBM) approach on single cell evaluation pairs.

Training Dataset	Predicting Dataset	scDHA	XGBoost	RF	DL	GBM
Baron (Human)	Segerstolpe	0.93	0.82	0.32	0.60	0.39
Baron (Human)	Muraro	0.88	0.86	0.79	0.72	0.74
Baron (Human)	Xin	0.99	0.93	0.49	0.03	0.84
Baron (Human)	Wang	0.96	0.27	0.28	0.01	0.60
Segerstolpe	Baron (Human)	0.94	0.83	0.71	0.21	0.49
Segerstolpe	Muraro	0.96	0.81	0.88	0.73	0.74
Segerstolpe	Xin	0.99	1	0.97	0.46	0.99
Segerstolpe	Wang	0.99	0.98	0.93	0.22	0.97
Xin	Baron (Human)	0.99	0.55	0.60	0.77	0.46
Xin	Segerstolpe	0.99	0.98	0.91	0.78	0.92
Xin	Muraro	0.97	0.70	0.82	0.57	0.42
Xin	Wang	1	1	0.58	0.58	0.96
Muraro	Baron (Human)	0.93	0.86	0.78	0.16	0.85
Muraro	Segerstolpe	0.97	0.93	0.65	0.65	0.72
Muraro	Xin	0.99	0.88	0.89	0.06	0.84
Muraro	Wang	0.98	0.85	0.64	0.01	0.73
Wang	Baron (Human)	0.93	0.14	0.38	0.30	0.38
Wang	Segerstolpe	0.92	0.90	0.75	0.44	0.91
Wang	Muraro	0.89	0.13	0.55	0.46	0.52
Wang	Xin	0.97	1	0.90	0.76	0.96

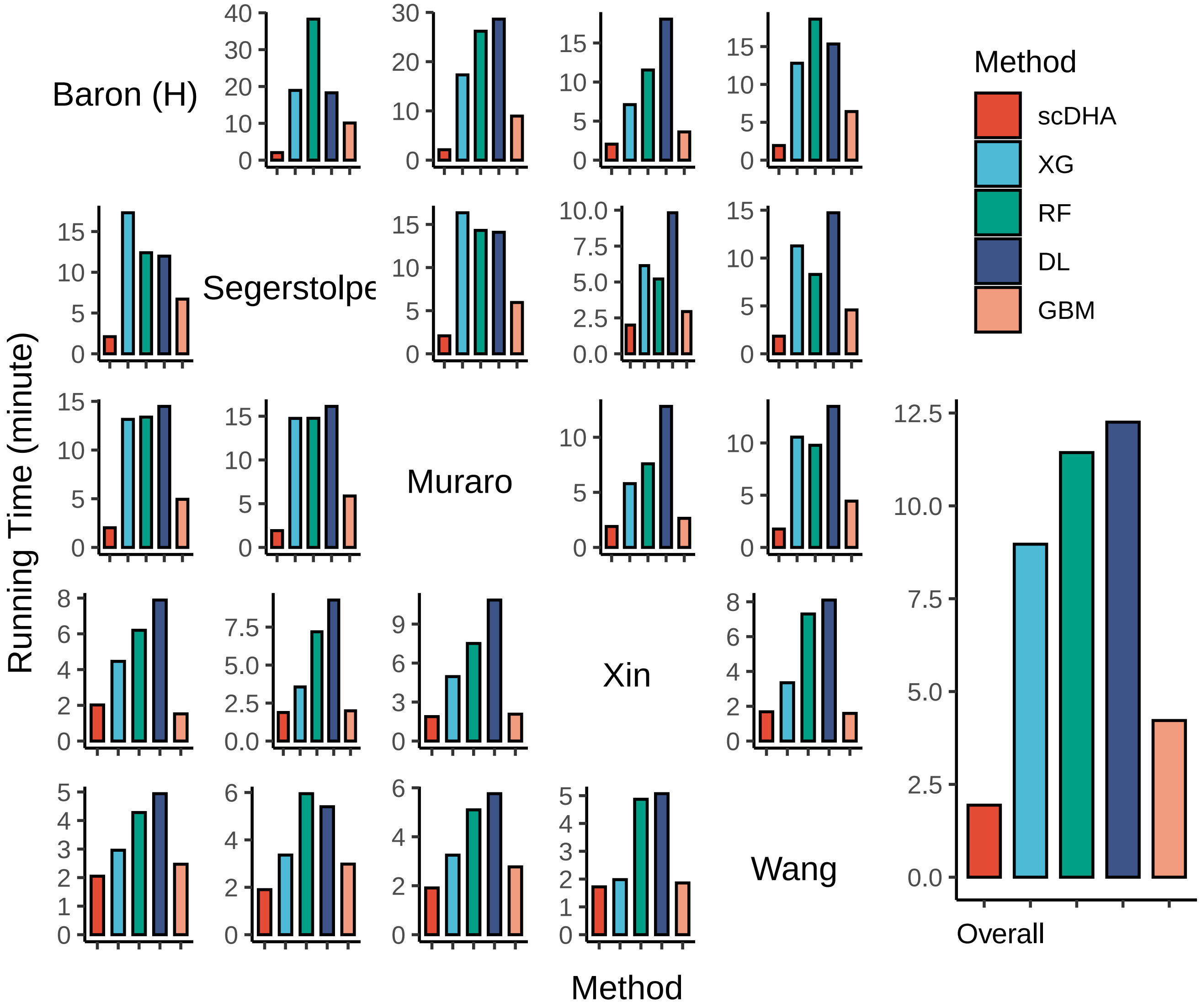

Supplementary Figure 20. Running time of scDHA, XGBoost, Random Forest (RF), Deep Learning (DL), Gradient Boosted Machine (GBM) using five human pancreatic datasets. In each scenario (row), we use one dataset as training and the rest as testing, resulting in 20 train-predict pairs.

4 Supplementary Note 4: Time trajectory inference

We compare scDHA with Monocle[25], TSCAN[26], Slingshot[27], and SCANPY. The pseudo-temporal ordering of the three mouse embryo datasets, Yan, Goolam, and Deng, are shown in Supplementary Figure 21. The time trajectories inferred by each method are shown in Supplementary Figure 22.

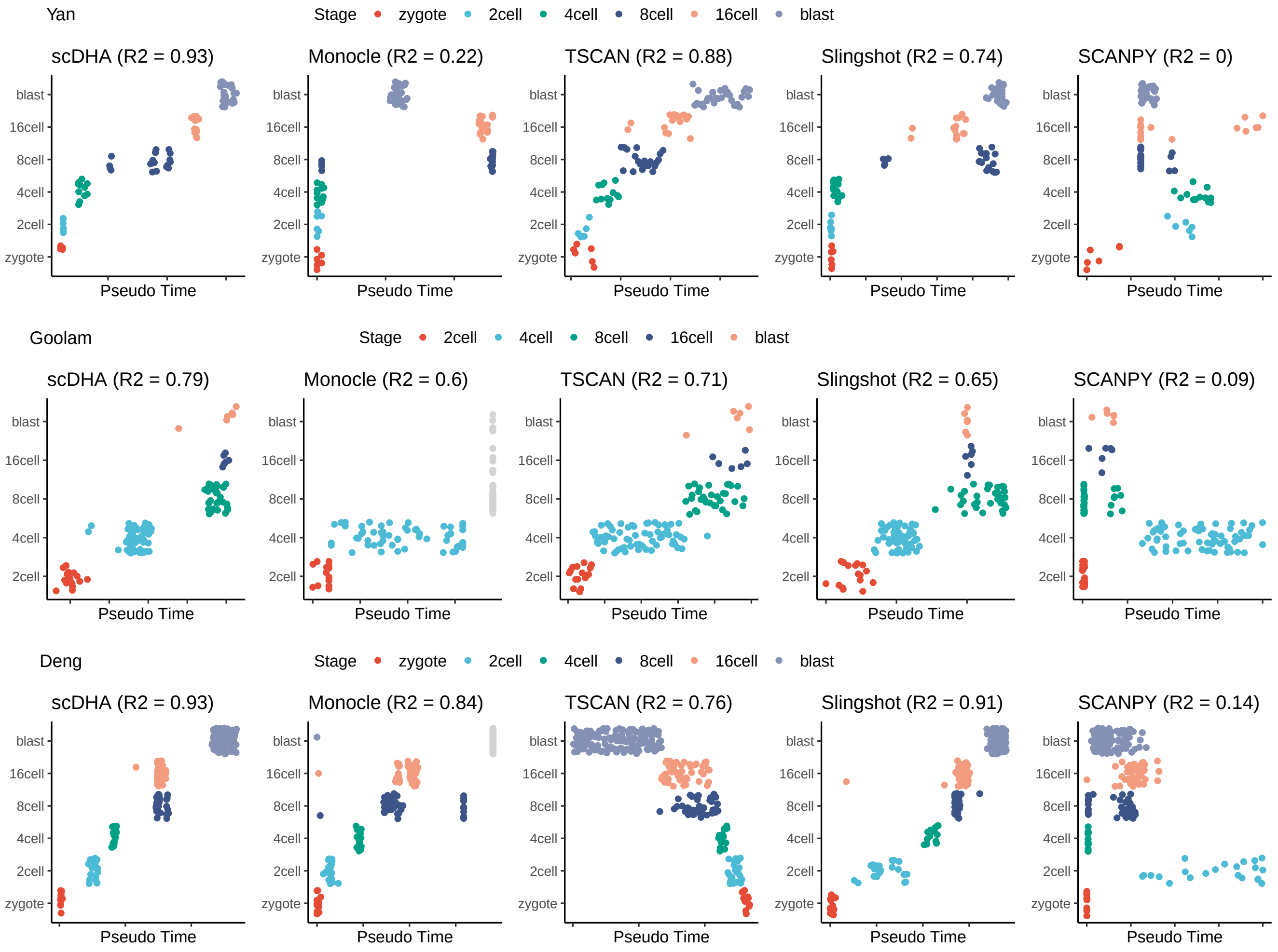

Supplementary Figure 21. Pseudo-time inferred by scDHA, Monocle, TSCAN, Slingshot, and SCANPY for the Yan, Goolam, and Deng datasets. R-squared values shown in each panel represent the correlation between the true developmental stages and inferred pseudo-time. Points with gray color indicate cells with infinite pseudo-time.

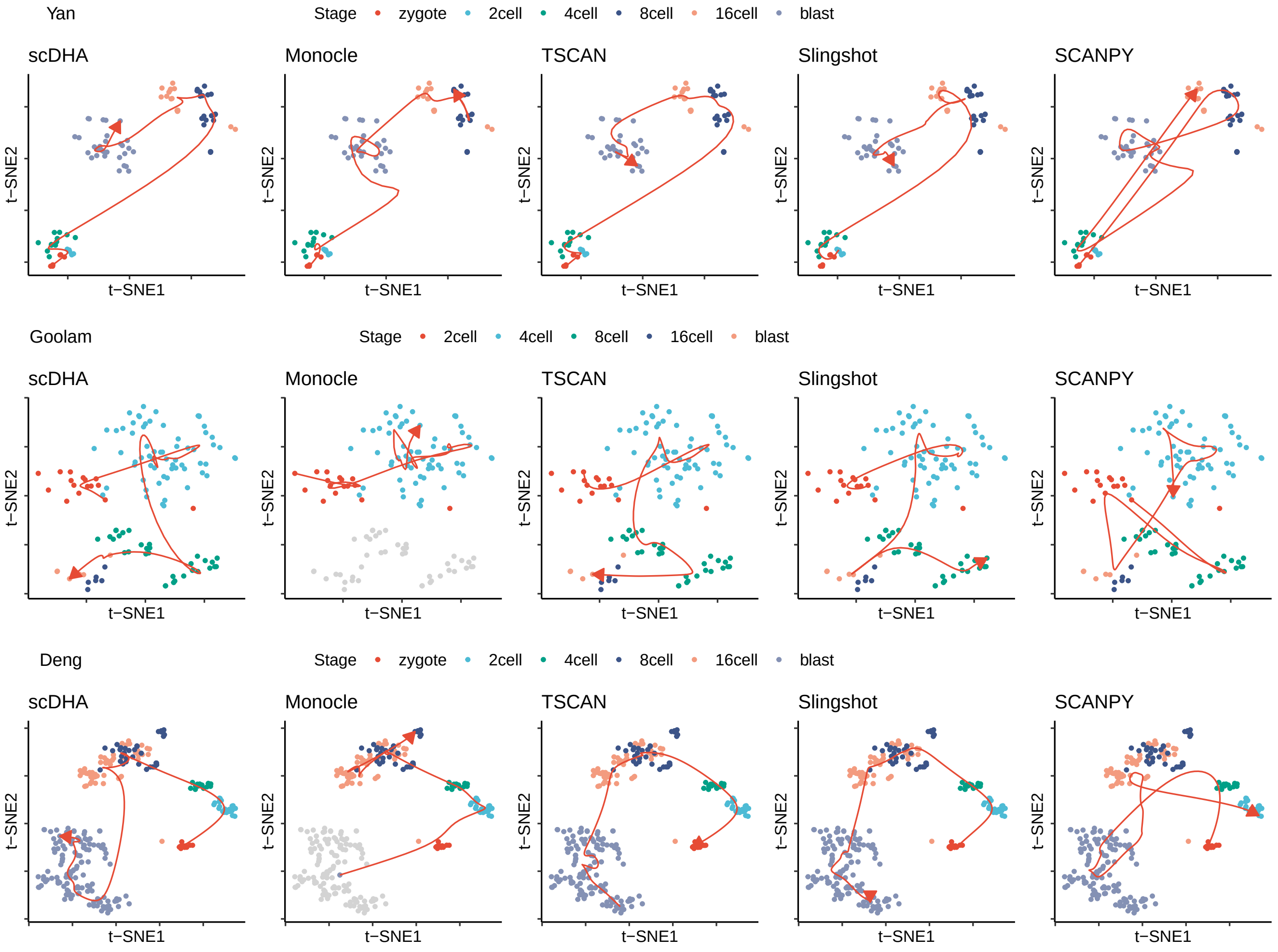

Supplementary Figure 22. Visualized trajectory inferred from Yan, Goolam, and Deng dataset using scDHA, Monocle, TSCAN, Slingshot, and SCANPY. Points with gray color mean cells with infinity pseudo time from Monocle.

5 Supplementary Note 5: Effects of data platforms on single-cell analysis

In 34 datasets analyzed, there are 19 plate-based datasets (Fluidigm C1, Tang, SMARTer, Smart-Seq1/2, CEL-seq2, STRT-Seq) and 15 flow-cell-based datasets (inDrop, Drop-seq, 10X Genomics). There are 4 platforms that have more than 5 datasets per platform: Smart-Seq1/2, SMARTer, inDrop, and 10X Genomics. We compared scDHA with other methods for the six protocol groups: plate-based (19 datasets), flow-cell-based (15 datasets), Smart-Seq1/2 (6 datasets), SMARTer (8 datasets), inDrop (5 datasets), and 10X Genomics (6 datasets).

Supplementary Figure 23 shows the performance of the clustering methods across the 6 platform groups. scDHA is the only method that performs consistently well across all six platform groups. The average ARI values of scDHA are close to 0.8 in all 6 groups. In contrast, the ARI values of other methods greatly differ across the platform groups. The average ARI of all methods drop when analyzing 10X Genomics data. This is partially due to the high dropout rate of 10X Genomics (the average dropout rates of Smart-Seq1/2, SMARTer, inDrop, and 10X Genomics datasets are 72.47, 76.61, 87.55, 91.50, respectively).

Supplementary Figure 24 shows the performance of the visualization methods. The silhouette values of all methods change across the platform groups. However, scDHA consistently outperforms other methods in each platform. Similar to clustering, the performance of all methods dropped when analyzing 10x Genomics.

For pseudo-time inference, as described in the main text, we analyzed only three datasets: Yan (Tang), Goolam (Smart-Seq2), Deng (Smart-Seq2). Similarly, classification only involves five datasets: Baron (inDrop), Segerstolpe (Smart-Seq2), Muraro (CEL-Seq2), Xin (SMARTer), and Wang (SMARTer). We did not compare the platforms due to the low number of datasets in each platform.

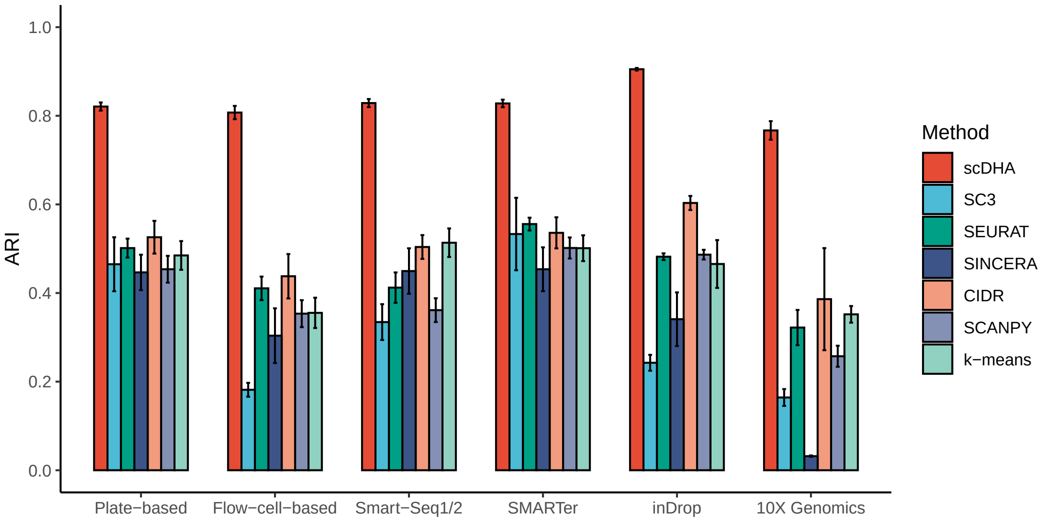

Supplementary Figure 23. Clustering performance of scDHA, SC3, SEURAT, SINCERA, CIDR, SCANPY, and k-means across six data platforms. Data are presented as mean values +/- variance

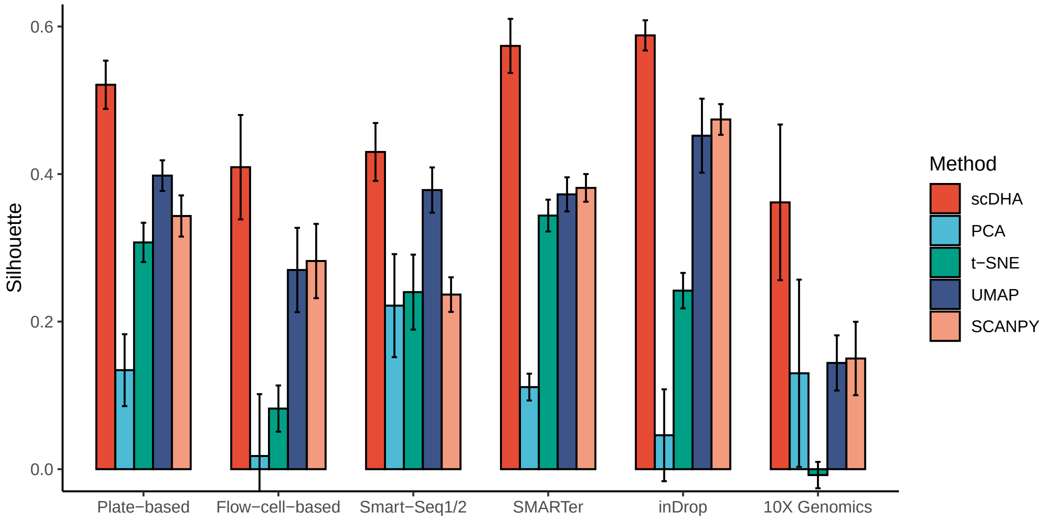

Supplementary Figure 24. Average silhouette values obtained from 2D representations across six data platforms. Data are presented as mean values +/- variance

6 Supplementary Note 6: Impact of the hierarchical autoencoder

scDHA provides a complete analysis pipeline. It is different from existing autoencoders, such as scVI[28], that were developed for dimension reduction or imputation.

Regarding capability, the scDHA package provides a complete analysis pipeline from feature selection (first module, non-negative kernel autoencoder) to dimension reduction (second module, VAE) and downstream analyses (visualization, clustering, classification, and pseudo-time inference). In contrast, scVI focuses on dimension reduction and data imputation. The output of scVI consists of a low-dimensional representation and the imputed data. The scVI package itself is not capable of clustering, visualization, classification, and pseudo-time inference.

Regarding implementation of the autoencoder, there are two key differences between scDHA and scVI. The first difference is that scDHA implemented a hierarchical autoencoder that consists of two modules: the first autoencoder to remove noise, and the second autoencoder to compress data (VAE is part of the second module). The first module filters out the noisy features and thus improves the quality of the data. Also, smaller number of features leads to a reduced time complexity in the second module and downstream analyses. The second difference is that we added an additional step to the variational autoencoder (in the second module) to generate multiple realizations of the input. This step makes the VAE more robust.

To demonstrate the efficiency of the hierarchical autoencoder, we compare the performance of scDHA with scVI in too scenarios. In the first scenario, we provide a direct comparison between scVI and scDHA, using the same analysis methods (clustering, visualization, classification, and pseudo-time inference) implemented in scDHA to compressed data produced by each autoencoder. In the second scenario, we compare scDHA against scVI used in conjunction with third-party analysis methods (clustering, visualization, classification, and pseudo-time inference). In any scenario, scDHA has a better accuracy.

In the first scenario, we compare scVI with three versions of scDHA: the original scDHA (hierarchical autoencoder) and the two simplified versions of scDHA in which one of the two autoencoders was removed. We use the same analysis methods implemented in scDHA to analyze the compressed data generated by each autoencoder. Supplementary Figure 25 shows the performance of the four methods (scVI, scDHA, first module, second module) in clustering analysis. Each of the two autoencoders of scDHA, when used separately, did not perform as well as the hierarchical autoencoder. The original scDHA has an average ARI value of 0.81 whereas the first and second module have average ARI values of 0.51 and 0.73, respectively. As we explained above, the second module of scDHA is similar to scVI except that the second module has an additional step of multiple realizations. This extra step makes VAE more robust. Indeed, the second module has a higher average ARI value than scVI (0.73 compared to 0.64).

Supplementary Figure 26 shows the silhouette values obtained from the 2D visualizations of the 34 datasets. Similar to clustering performance, the hierarchical autoencoder outperforms the rest. The original scDHA has an average silhouette value of 0.47 whereas the first module, second module, and scVI have silhouette values of -0.1, 0.36, and 0.34, respectively. Again, the second module has a higher silhouette value than scVI (0.36 compared to 0.34) because of the additional step of multiple realizations.

Supplementary Figure 27 shows the classification accuracy of the methods using five human pancreas datasets. In each analysis scenario, we use one dataset as training and the rest as testing, resulting in 20 train-predict pairs for each method. The original scDHA has an average accuracy of 0.96 whereas the first module, second module, and scVI have accuracy values of 0.57, 0.88, and 0.79, respectively. The second module has a higher average accuracy than scVI (0.88 compared to 0.79).

Supplementary Figure 28 shows the R-squared values obtained from pseudo-time trajectory of the three datasets, Yan, Goolam, and Deng. The original scDHA has the highest R-squared values (0.88 compared to 0.77, 0.84, and 0.60 of the first module, second module, and scVI, respectively). In this

analysis, all three scDHA autoencoders have higher R-squared values than scVI.

Regarding the improvement of scDHA over other downstream analysis methods (clustering, visualization, classification, time trajectory), the major contributor is the hierarchical autoencoder. For example, scDHA clustering in conjunction with scVI has an average ARI of 0.64. This value is higher than the ARI of CIDR (which is 0.5), but it is not as high as scDHA clustering with hierarchical autoencoder (which is 0.81). For visualization, scDHA visualization with scVI has a silhouette value of 0.34. This value is similar to that of UMAP (which is 0.36) but is much lower than scDHA visualization with hierarchical autoencoder (which is 0.47). Regarding classification, the accuracy of scDHA classification with scVI is 0.79. This value is close to that of XGBoost (which is 0.77) and is much lower than the hierarchical scDHA (which is 0.96). Regarding time trajectory, scDHA inference with scVI has an average R-squared of 0.60, which is lower than all other methods: Monocle, TSCAN, Slinghot, and scDHA inference with three scDHA autoencoders.

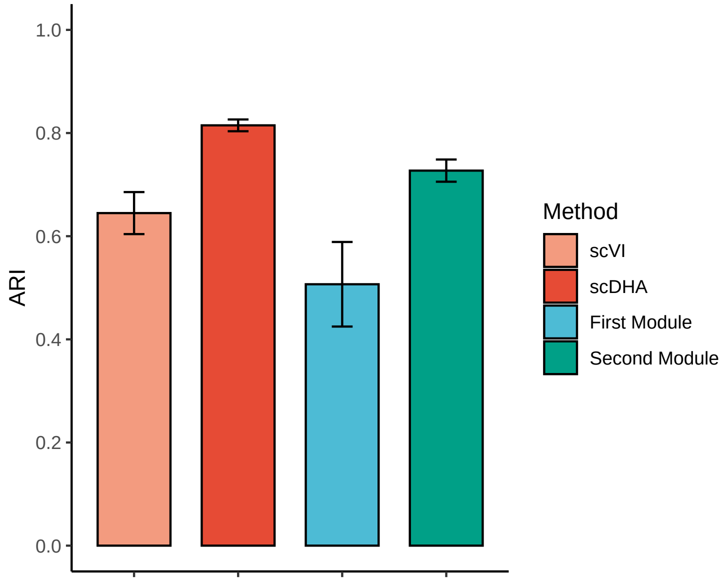

Supplementary Figure 25. Clustering performance of the four methods, scVI, scDHA, and the two simplified versions of scDHA, on 34 single-cell datasets. Data are presented as mean values +/- variance. Overall, scDHA (hierarchical autoencoder), as well as its second module (variational autoencoder with multiple realizations), has higher ARI values than scVI (variational autoencoder).

In the second scenario, we use scVI to impute the data and then then analyze the imputed data using existing methods. Supplementary Figure 29 shows the ARI values obtained from clustering results. scVI improves the performance of SEURAT, SINCERA, and CIDR but worsens the performance of SC3, SCANPY, and k-means. scDHA outperforms all other methods, with and without scVI, by having the highest average ARI values.

Supplementary Figure 30 shows the silhouette values obtained from 2D representations of the 34 datasets. Interestingly, the performance of all four methods, PCA, t-SNE, UMAP, and SCANPY, drops when used with scVI. scDHA outperforms other methods, with and without scVI, by having the highest silhouette values.

Supplementary Figure 31 shows the accuracy of the classification methods. scVI improves the accuracy of deep learning (DL) but worsens the performance of the XGBoost (XGB), Random Forest (FR), and Gradient Boosted Machine (GBM). Interestingly, all four methods have comparable accuracy when used

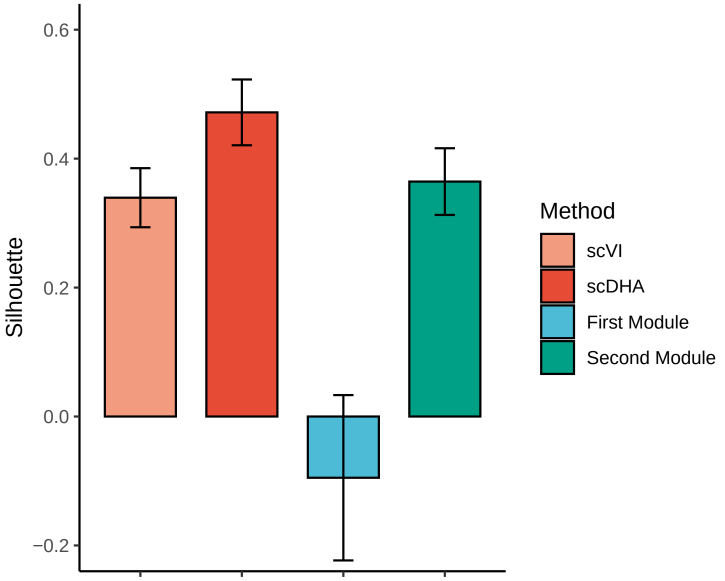

Supplementary Figure 26. Average silhouette values obtained from the 2D visualizations of 34 single-cell datasets using scVI, scDHA, and the two simplified versions of scDHA. Data are presented as mean values +/- variance. Overall, scDHA (hierarchical autoencoder), as well as its second module (variational autoencoder with multiple realizations), has higher silhouette values than scVI (variational autoencoder).

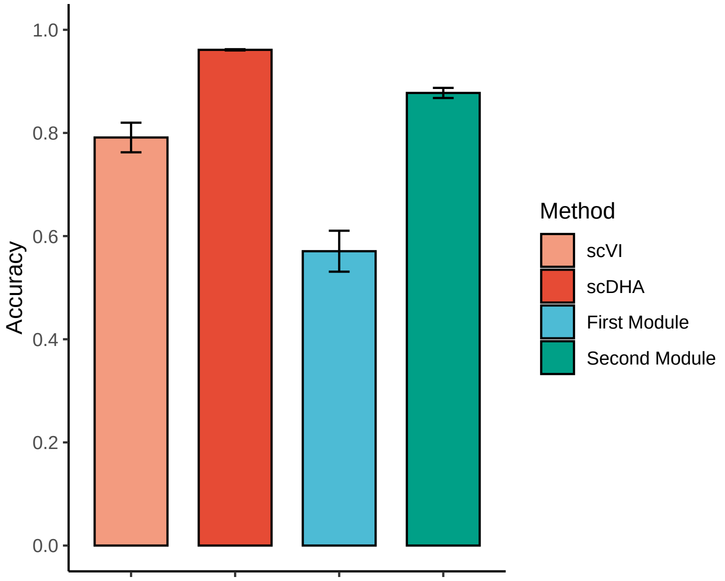

Supplementary Figure 27. Classification accuracy of the four methods, scVI, scDHA, and the two simplified versions of scDHA, on 5 human pancreas datasets. In each analysis scenario, we use one dataset as training and the rest as testing, resulting in 20 train-predict pairs for each method. Data are presented as mean values +/- variance. Overall, scDHA (hierarchical autoencoder), as well as its second module (variational autoencoder with multiple realizations), has higher accuracy than scVI (variational autoencoder).

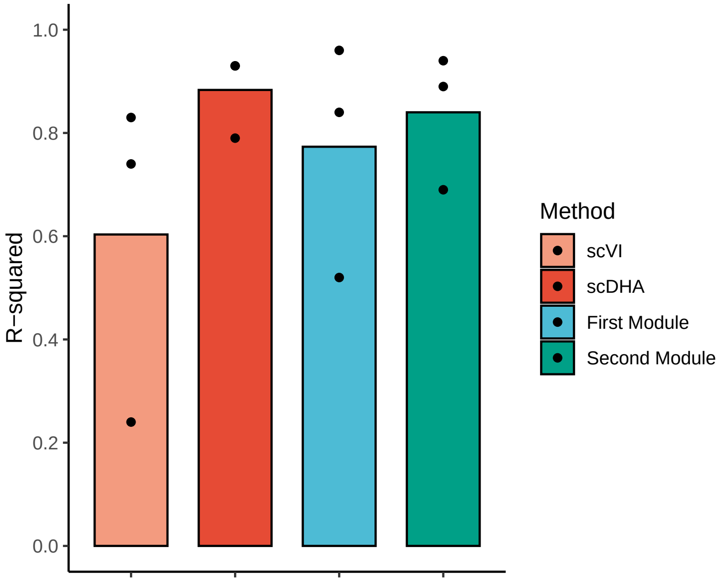

Supplementary Figure 28. R-squared values obtained from pseudo-time inference of three datasets (Yan, Goolam, and Deng) using the four methods, scVI, scDHA, and the two simplified versions of scDHA. Each point represents the R-squared value for a single dataset. Overall, all of the three scDHA methods have higher R-squared than scVI.

with scVI. Nevertheless, scDHA has higher accuracy than other methods, with and without scVI.

Finally, Supplementary Figure 32 shows the R-squared calculated from pseudo-time inference methods. scVI increases the R-squared values of Slinghot and SCANPY but decreases the R-squared of Monocle and TSCAN. scDHA has higher R-squared values in both cases.

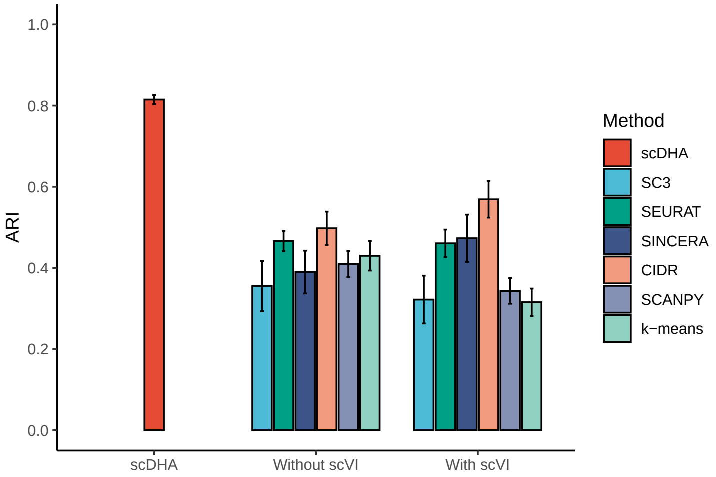

Supplementary Figure 29. Clustering performance of scDHA and 12 clustering methods (SC3, SEURAT, SINCERA, CIDR, SCANPY, and k-means with and without scVI) on 34 single-cell datasets. Data are presented as mean values +/- variance.

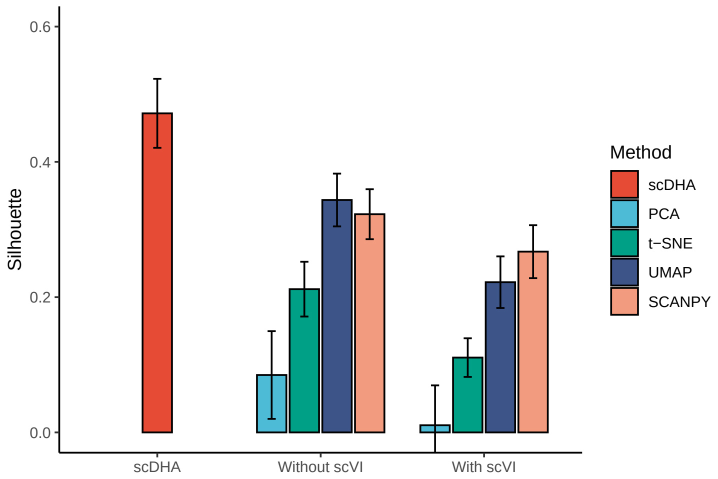

Supplementary Figure 30. Silhouette values obtained from 2D representations using scDHA and eight visualization methods (PCA, t-SNE, UMAP, and SCANPY with and without scVI) on 34 single-cell datasets. Data are presented as mean values +/- variance.

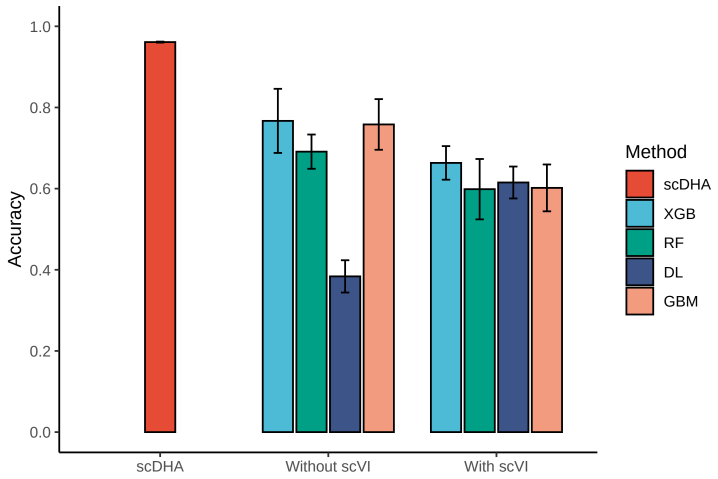

Supplementary Figure 31. Classification accuracy of scDHA and eight classification methods (XGBoost, Random Forest, Deep Learning, Gradient Boosted Machine with and without scVI) on 20 train-predict pairs. Data are presented as mean values +/- variance.

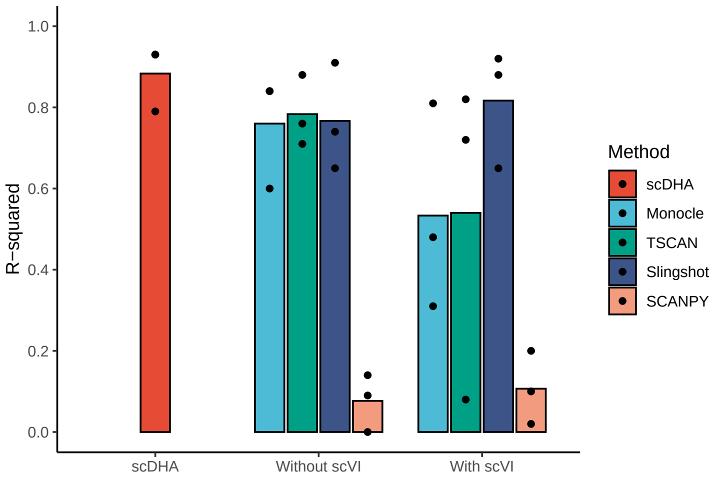

Supplementary Figure 32. R-squared values obtained from scDHA and eight pseudo-time inference methods (Monocle, TSCAN, Slingshot, and SCANPY with and without scVI). Each point represents the R-squared value for a single dataset.

References

1. Rand, W. M. Objective criteria for the evaluation of clustering methods. *Journal of the American Statistical Association* **66**, 846–850 (1971).
2. Hubert, L. & Arabie, P. Comparing partitions. *Journal of Classification* **2**, 193–218 (1985).
3. Kiselev, V. Y. *et al.* SC3: consensus clustering of single-cell RNA-seq data. *Nature Methods* **14**, 483–486 (2017).
4. Satija, R., Farrell, J. A., Gennert, D., Schier, A. F. & Regev, A. Spatial reconstruction of single-cell gene expression data. *Nature Biotechnology* **33**, 495–502 (2015).
5. Guo, M., Wang, H., Potter, S. S., Whitsett, J. A. & Xu, Y. SINCERA: A pipeline for single-cell RNA-seq profiling analysis. *PLOS Computational Biology* **11**, e1004575 (2015).
6. Lin, P., Troup, M. & Ho, J. W. K. CIDR: Ultrafast and accurate clustering through imputation for single-cell RNA-seq data. *Genome Biology* **18**, 59 (2017).
7. Zappia, L., Phipson, B. & Oshlack, A. Splatter: Simulation of single-cell RNA sequencing data. *Genome Biology* **18**, 1–15 (2017).
8. Arisdakessian, C., Poirion, O., Yunits, B., Zhu, X. & Garmire, L. X. DeepImpute: an accurate, fast, and scalable deep neural network method to impute single-cell RNA-seq data. *Genome Biology* **20**, 1–14 (2019).
9. Tsoucas, D. *et al.* Accurate estimation of cell-type composition from gene expression data. *Nature Communications* **10**, 2975 (2019).
10. Eraslan, G., Simon, L. M., Mircea, M., Mueller, N. S. & Theis, F. J. Single-cell rna-seq denoising using a deep count autoencoder. *Nature Communications* **10**, 390 (2019).
11. Tran, H. T. N. *et al.* A benchmark of batch-effect correction methods for single-cell RNA sequencing data. *Genome Biology* **21**, 12 (2020).
12. Saelens, W., Cannoodt, R., Todorov, H. & Saeys, Y. A comparison of single-cell trajectory inference methods. *Nature Biotechnology* **37**, 547–554 (2019).
13. He, K., Zhang, X., Ren, S. & Sun, J. Deep residual learning for image recognition. In *2016 IEEE Conference on Computer Vision and Pattern Recognition (CVPR)*, 770–778 (2016).
14. Huang, G., Liu, Z., Van Der Maaten, L. & Weinberger, K. Q. Densely connected convolutional networks. In *2017 IEEE Conference on Computer Vision and Pattern Recognition (CVPR)*, 4700–4708 (2017).
15. Szegedy, C. *et al.* Going deeper with convolutions. In *2015 IEEE Conference on Computer Vision and Pattern Recognition (CVPR)*, 1–9 (2015).
16. Tan, M. & Le, Q. EfficientNet: Rethinking model scaling for convolutional neural networks. In *Proceedings of the 36th International Conference on Machine Learning*, vol. 97, 6105–6114 (Long Beach, California, USA, 2019).
17. Amir, E.-a. D. *et al.* viSNE enables visualization of high dimensional single-cell data and reveals phenotypic heterogeneity of leukemia. *Nature Biotechnology* **31**, 545 (2013).
18. Maaten, L. v. d. & Hinton, G. Visualizing data using t-SNE. *Journal of Machine Learning Research* **9**, 2579–2605 (2008).
19. Becht, E. *et al.* Dimensionality reduction for visualizing single-cell data using UMAP. *Nature Biotechnology* **37**, 38–44 (2019).
20. McInnes, L., Healy, J. & Melville, J. Umap: uniform manifold approximation and projection for dimension reduction. *arXiv preprint arXiv:1802.03426* (2018).
21. Chen, T. & Guestrin, C. XGBoost: A scalable tree boosting system. In *Proceedings of the 22Nd ACM SIGKDD International Conference on Knowledge Discovery and Data Mining*, KDD '16, 785–794

(ACM, New York, NY, USA, 2016).
22. Breiman, L. Random Forests. *Machine Learning* **45**, 5–32 (2001).
23. LeCun, Y., Bengio, Y. & Hinton, G. Deep learning. *Nature* **521**, 436–444 (2015).
24. Friedman, J. H. Greedy function approximation: A Gradient Boosting Machine. *The Annals of Statistics* **29**, 1189–1232 (2001).
25. Trapnell, C. *et al.* The dynamics and regulators of cell fate decisions are revealed by pseudotemporal ordering of single cells. *Nature Biotechnology* **32**, 381–386 (2014).
26. Ji, Z. & Ji, H. TSCAN: Pseudo-time reconstruction and evaluation in single-cell RNA-seq analysis. *Nucleic Acids Research* **44**, e117–e117 (2016).
27. Street, K. *et al.* Slingshot: cell lineage and pseudotime inference for single-cell transcriptomics. *BMC Genomics* **19**, 477 (2018).
28. Lopez, R., Regier, J., Cole, M. B., Jordan, M. I. & Yosef, N. Deep generative modeling for single-cell transcriptomics. *Nature Methods* **15**, 1053–1058 (2018).
